# Rediscovery and morpho-molecular characterization of three astome ciliates, with new insights into eco-evolutionary associations of astomes with their annelid hosts

**DOI:** 10.1007/s42995-024-00275-5

**Published:** 2025-03-17

**Authors:** Tomáš Obert, Tengyue Zhang, Ivan Rurik, Peter Vďačný

**Affiliations:** 1 Department of Zoology, Faculty of Natural Sciences, Comenius University in Bratislava, 842 15 Bratislava, Slovak Republic; 2 The Key Laboratory of Zoological Systematics and Application, College of Life Sciences, Hebei University, Baoding, 071002 China

**Keywords:** Comparative network analyses, Endosymbionts, Integrative taxonomy, Oligochaeta, Oligohymenophorea, Phylogeny

## Abstract

Astome ciliates live in the digestive tract of a broad spectrum of marine, freshwater, and terricolous annelids. In aquatic lumbriculid and criodrilid oligochaetes collected in Central Europe, we rediscovered three insufficiently known astomes: *Hoplitophrya secans*, *Mesnilella clavata*, and *Buchneriella criodrili*. Their morphology was studied using in vivo observation, protargol, and dry silver nitrate impregnation. Multiple nuclear and mitochondrial molecular markers were used to determine their phylogenetic positions and reconstruct their evolutionary history. According to our phylogenetic analyses: (1) mouthless ciliates isolated from annelids form a robustly supported monophylum within the class Oligohymenophorea, (2) the progenitor of astomes invaded the digestive tract of marine polychaetes during the Paleozoic era, (3) lumbricid earthworms likely served as a source of astomes for criodrilid, almid, and megascolecid earthworms, (4) the ancestral host of the earthworm-dwelling astome clade led an endogeic lifestyle, and (5) there were multiple independent transfers of astomes from endogeic to epigeic and anecic earthworms. These findings support previous views of the annelid phylogeny, suggesting that astomes reside and evolve in tandem with annelids for several hundred million years.

**Supplementary Information:**

The online version contains supplementary material available at 10.1007/s42995-024-00275-5.

## Introduction

The phylum Ciliophora Doflein, 1901 (ciliates) is one of the most diverse clades of eukaryotic microorganisms, with a long evolutionary history dating back to the Proterozoic period more than a billion years ago (Jiang et al. 2024; Lynn 2008; Parfrey et al. 2011). Over geological time, approximately one-third of ciliate species have become symbionts of animals, ranging from platyhelminths, annelids, mollusks, and arthropods through echinoderms to both poikilo- and homoiotherm vertebrates (for reviews, see Corliss 2000 and Lynn 2008). Within invertebrate hosts, annelids harbor a diverse ciliate assemblage comprising four groups: the family Plagiotomidae from the class Spirotrichea (Obert and Vďačný 2020a; Obert et al. 2022), the family Nyctotheridae from the class Armophorea (Albaret and Njiné 1975; Fokam et al. 2014; Ngassam 1983a), the family Hysterocinetidae (Obert et al. 2023; Raabe 1972) and the subclass Astomatia (de Puytorac 1954, 1969, 1972) both from the class Oligohymenophorea. According to molecular clock analyses, ciliates first populated the digestive tract of annelids in the Paleozoic period, between 587 and 247 million years ago (Zhang and Vďačný 2022). Further independent colonizations of the annelid mid- and hindgut occurred more recently, e.g., in the Cenozoic about 35 million years ago (Obert et al. 2022).

**Fig. 1:**
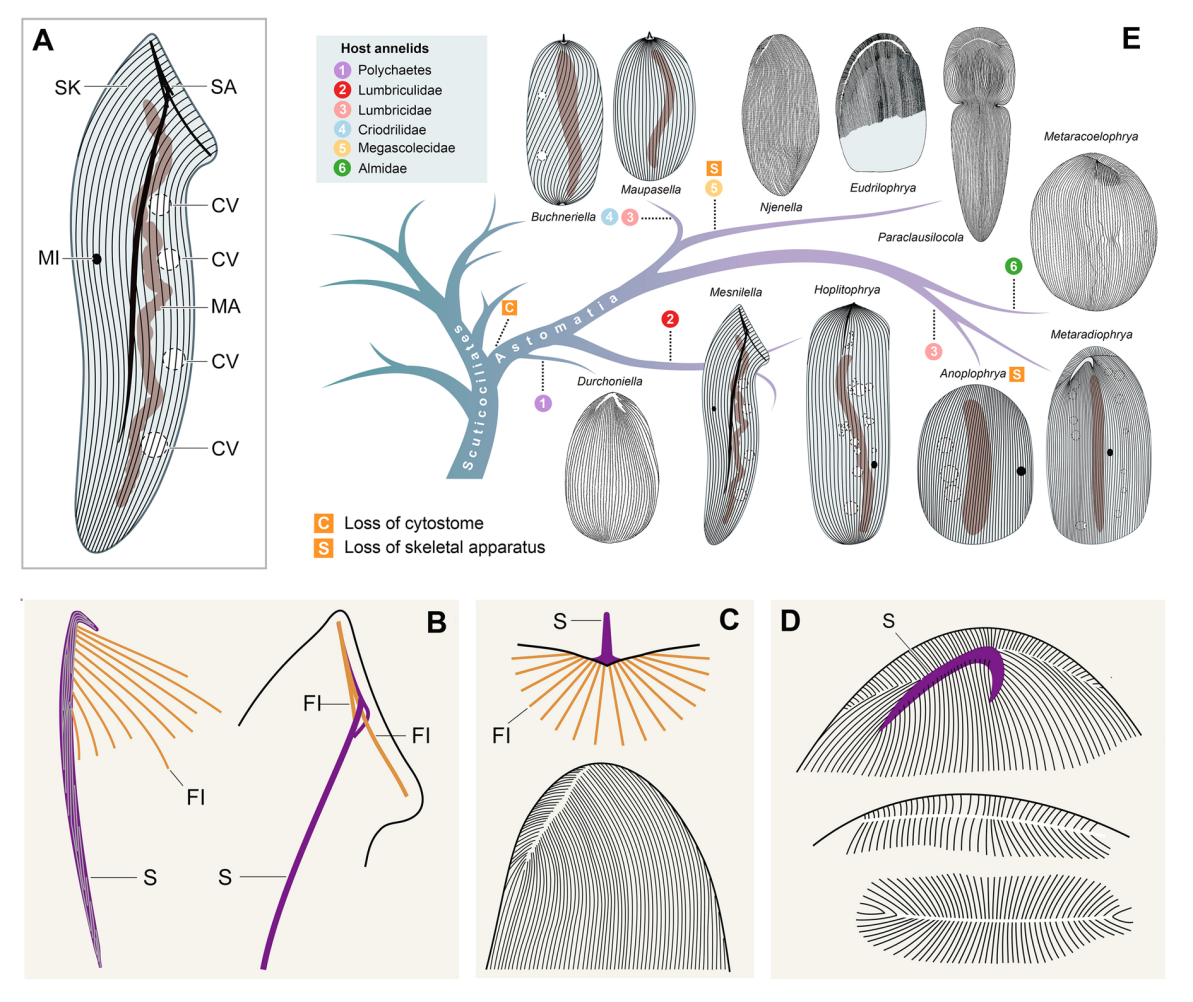
Introduction to morphological characters (**A**‒**D**) and phylogenetic relationships of astome ciliates (**E**). **A** A typical astome to show the key morphological structures. **B** Skeletal apparatus of two representatives of the family Hoplitophryidae. **C** Skeletal apparatus of a representative of the family Maupasellidae (upper part) and ciliary pattern in the anterior body portion of the genus *Njinella* (lower part), which completely lost the skeletal apparatus*.* **D** Skeletal apparatus and ciliary pattern in the anterior body portion of the genus *Metaradiophrya* (left) as well as the anterior and posterior sutures of the closely related genus *Anoplophrya* (right), which completely lost the skeletal apparatus. **E** The subclass Astomatia stemmed within the paraphyletic subclass Scuticociliatia. Astomes living in terrestrial and freshwater oligochaetes (Almidae, Criodrilidae, Lumbriculidae, Lumbricidae, and Megascolecidae) branched off after astomes inhabiting marine polychaetes. *Durchodniella* from Sauvadet et al. (2017), *Eudrilophrya*, *Metaracoelophrya* and *Paraclausilocola* from Fokam (2012), and *Njinella* from Ngassam (1983b). *CV* contractile vacuoles, *FI* fibers, *MA* macronucleus, *MI* micronucleus, *S* spicule, *SA* skeletal apparatus, *SK* somatic kineties

The subclass Astomatia is the most diverse among the four ciliate groups populating the annelid digestive tract, with over two hundred species from more than 50 genera (Lynn 2008). These ciliates have lost their oral ciliature and cytostome (cell mouth), as their name implies. They typically attach to the intestinal epithelium by a variety of holdfast and skeletal organelles located on the anterior pole or in the anterior region of the cell (Fig. 1A‒D), although the skeletal attachment apparatus has been lost multiple times (Fig. 1E). Astomes range from < 80 µm to > 1 mm (de Puytorac 1954, 1969, 1972), with varying cell shapes (Fig. 1E). The macronucleus is elongate, often almost as long as the cell, and the micronucleus is globular, ellipsoidal, or fusiform (Fig. 1A, E). Contractile vacuoles are arranged in one or two rows, form a stripe, or are scattered throughout the cell (Fig. 1A, E). Finally, the somatic ciliation is usually dense, holotrichous, and formed by monokinetids arranged in meridional or spiraling rows often creating anterior and posterior sutures (Fig. 1C, D).

Annelid hosts of astomes live in marine, freshwater, and terrestrial environments. Terricolous earthworms are subdivided into three ecological groups (Jeffery et al. 2010): epigeic (litter- or surface-dwelling), anecic (topsoil-dwelling), and endogeic (subsoil-dwelling). Phylogenetic studies suggested that astome ciliates cluster according to associations with higher taxa of their hosts and/or their ecological groups (Fig. 1E; Fokam et al. 2011; Obert and Vďačný 2019, 2020b, 2021; Obert et al. 2021). Astomes isolated from terrestrial megadrilid oligochaetes form a sister group to astomes from marine polychaetes (Sauvadet et al. 2017), or astomes inhabiting polychaetes are placed more basally than astomes living in oligochaetes (Park and Leander 2024). Astomes inhabiting the digestive tract of megascolecid and almid earthworms form distinct and well-separated clades (Fokam et al. 2011). Astomes living in endogeic earthworms form deep-branching lineages, while astomes from epigeic and anecic earthworms emerged multiple times independently in the terminal radiation of the subclass Astomatia (Obert and Vďačný 2019, 2020b, 2021; Obert et al. 2021). This eco-evolutionary pattern mirrors the fact that the endogeic lifestyle (subsoil-dwelling and geophagy) represents the ground pattern of lumbricid earthworms, and epigeic and anecic earthworms evolved several times convergently from endogeic ancestors (Domínguez et al. 2015). However, after the inclusion of further astomes in phylogenetic analyses, it seems that ciliates isolated from terrestrial and semi-aquatic lumbricid earthworms form a paraphyletic assemblage comprising also astomes from megascolecid and almid earthworms (Obert et al. 2021). It, therefore, seems that further analysis of astomes is needed to assess their phylogeny.

The morphological and molecular diversity of astome ciliates associated with soil earthworms of the family Lumbricidae have been investigated in detail (Obert and Vďačný 2019, 2020b, 2021; Obert et al. 2021). However, the taxonomy and phylogeny of astomes living in freshwater oligochaetes is still poorly understood. To better understand the eco-evolutionary associations of ciliates and annelids, we selected two aquatic oligochaetes: one representing an old lineage (*Lumbriculus variegatus*, Lumbriculidae) and one representing a more recent lineage (*Criodrilus lacuum*, Criodrilidae). By necessity, due to questionable taxonomic reports, we have also provided a historical and current reassessment of the ciliate taxa (*Hoplitophrya secans*, *Mesnilella clavata*, and *Buchneriella criodrili*) isolated from the worms. By examining astomes from these oligochaetes, we were able to test the following hypotheses: (1) astomes first invaded marine polychaetes, then freshwater lumbriculid oligochaetes, and finally terrestrial and aquatic earthworms and (2) endogeic lumbricids were the first terrestrial earthworms to be colonized.

## Material and methods

### Sampling and morphological taxonomic methods

The host oligochaetes, *L. variegatus* Müller, 1774 and *C. lacuum* Hoffmeister, 1845, were collected from three localities in western Slovakia, Central Europe (see ciliate descriptions for details). They were morphologically (Timm 2009) and molecularly identified using barcoding of the nuclear 18S rRNA gene, ITS region, partial 28S rRNA gene sequences as well as of the mitochondrial genes coding for NADH-ubiquinone oxidoreductase chain 1 (ND1) and cytochrome *c* oxidase subunit I (COI). Primers and PCR protocols used are described elsewhere (Obert et al. 2021).

The digestive tracts were dissected and examined for ciliates, which were isolated and identified in vivo at 50‒400 × and 1000 × (oil immersion) magnification, with bright field and DIC optics, following guidelines of Foissner (2014). The nuclear apparatus and somatic ciliary patterns were visualized with protargol staining (Wilbert 1975). The silverline system was revealed using dry silver nitrate impregnation (Klein 1958).

### Molecular methods

Ciliates were cleaned in Ringer’s solution (0.6% NaCl) and stored in 180 μL of cell lysis buffer (Promega, Fitchburg, Wisconsin, USA) at 6 °C. Each sample contained one cell (*n* = 5 cells for *H. secans* and *B. criodrili* and 9 for *M. clavata*). Genomic DNA was isolated using the ReliaPrep^™^ Blood gDNA Miniprep System (Promega, Fitchburg, Wisconsin, USA). Three nuclear rRNA genes (18S, 5.8S, 28S), their spacers (ITS1, ITS2), and the barcoding mitochondrial COI and 16S rRNA genes were PCR amplified, following the protocols of Obert et al. (2021). PCRs were conducted with GoTaq^®^ Long PCR Master Mix (Promega, Fitchburg, Wisconsin, USA). Sequences were acquired by bidirectional Sanger sequencing on an ABI 3730 automatic sequencer in Macrogen Europe B.V. (Amsterdam, The Netherlands).

### Phylogenetic tree-building methods

The quality of acquired sequences was checked in Chromas ver. 2.6.6 (Technelysium Pty Ltd, South Brisbane, Australia). High-quality sequences (Phred score ≥ 20) were assembled into contigs using BioEdit ver. 7.2.5 (Hall 1999). Individual genes were aligned on the MAFFT ver. 7 server (Katoh et al. 2019), using the E-INS-i strategy and the 200PAM/κ = 2 scoring matrix with a gap opening penalty at 1.53. No masking was employed.

Three datasets were assembled to determine the phylogenetic position of acquired sequences within the subclass Astomatia. The first dataset contained only 18S rRNA gene sequences sampled across the class Oligohymenophorea, with the subclass Peritrichia as an outgroup, following the study of Jiang et al. (2019). The second dataset consisted of sequences from the mitochondrial and nuclear rRNA genes and their spacers obtained from members of the subclass Astomatia. The third dataset comprised the mitochondrial and nuclear rRNA genes and their spacers plus the mitochondrial COI sequences. For GenBank accession numbers of sequences used in datasets 1‒3, see Supplementary Tables 1 and 2.

Phylogenetic trees were constructed using the maximum likelihood (ML) approach and Bayesian inference. ML trees were built in IQ-TREE ver. 1.6.12 (Nguyen et al. 2015) on the IQ-TREE web server (http://iqtree.cibiv.univie.ac.at/) (Trifinopoulos et al. 2016). The best evolutionary model was determined for each molecular marker using the built-in ModelFinder (Kalyaanamoorthy et al. 2017). The robustness of the inferred ML tree topologies was assessed by 1000 ultrafast bootstrap pseudoreplicates, and the bnni algorithm was used to avoid overestimating bootstrap support (Hoang et al. 2018). Bayesian analyses were conducted using MrBayes on XSEDE ver. 3.2.7a (Ronquist et al. 2012) on the CIPRES portal (http://www.phylo.org/) (Miller et al. 2010) with the following settings: (1) five million generations for the first and second datasets and two million generations for the third dataset; (2) a sampling frequency of trees and parameters at one hundred; (3) a relative burn-in fraction at 25%; and (4) prior parameters of the best evolutionary models (as determined by ModelFinder) were implemented with the ‘lset’ and ‘prset’ commands. The convergence of Bayesian analyses was considered successful since the standard deviation of split frequencies was consistently < 0.01, the average potential scale reduction factor was 1.00, and the effective sample sizes of all parameters were ≥ 200, with no obvious trends in the plots of generations versus log probability. All trees were visualized in FigTree ver. 1.2.3 (http://tree.bio.ed.ac.uk/software/figtree/).

Since phylogenetic positions of some astome taxa were poorly resolved, tree topology testing was conducted. In each alternative scenario, only the monophyly of/kinships within a certain group was forced; all other relationships were left unspecified. The best unconstraint ML tree and five constraint trees (Table 1) were constructed from the first dataset in IQ-TREE with the aforementioned settings. The reliability of alternative tree topologies was assessed using the approximately unbiased test (Shimodaira 2002), the weighted Shimodaira‒Hasegawa test (Shimodaira and Hasegawa 1999), the weighted Kishino‒Hasegawa test (Kishino and Hasegawa 1989), and 10,000 re-samplings using the RELL method (Kishino et al. 1990).

**Table 1:** Statistical tree topology tests to compare different evolutionary scenarios

Evolutionary scenario	logL	Δ logL	WKH	WSH	RELL	AU	Conclusion
Best scoring maximum likelihood tree (unconstrained)	‒ 21,807.8136	‒	0.578	0.961	0.294	0.777	‒
*Durchoniella* sister to *Pennarella*	‒ 21,814.7580	6.9444	0.091	0.217	0.029*	0.104	Rejected
Monophyly of astomes isolated from oligochaetes	‒ 21,810.7032	2.8896	0.220	0.567	0.108	0.351	Not rejected
(*Anoplophrya vulgaris*, ((*A. lumbrici*, *A. octolasionis*), (*A. allolobophorae*, *A. aporrectodeae*)))	‒ 21,812.2793	4.4657	0.291	0.536	0.137	0.329	Not rejected
(*Metaradiophrya varians*, (*M. speculorum*, (*M. lumbrici*, *M. chlorotica*)))	‒ 21,809.4902	1.6766	0.422	0.635	0.354	0.475	Not rejected
*Subanoplophrya* sister to all other astomes isolated from crassiclitellates	‒ 21,809.2686	1.4550	0.230	0.664	0.078	0.351	Not rejected

*logL* log likelihood, *Δ logL* difference between log likelihoods of a constrained and the best (unconstrained) tree, *WKH* weighted Kishino‒Hasegawa test, *WSH* weighted Shimodaira‒Hasegawa test, *RELL* bootstrap proportion using the RELL method, *AU* approximately unbiased test

*P < 0.05

### Comparative phylogenetic network methods

The first dataset was pruned to contain only astomes and the outgroup scuticociliates. Evolutionary scenarios that could not be rejected by tree topology testing served to build constrained time-calibrated trees. Unconstrained and constrained trees were constructed with BEAST ver. 2.6.7 (Bouckaert et al. 2014) using the GTR + I (= 0.5732) + Γ_4_ (= 0.4898) evolutionary model as selected by ModelFinder, relaxed lognormal clock, and the birth–death process model for the tree prior with lognormal birth and death rates. All other parameters were left as the default. A uniform distribution with a maximum age of 261 Ma (mega annum) and a minimum age of 200 Ma was assigned to the node uniting astomes inhabiting earthworms; this was based on molecular clock analyses of earthworms (Domínguez et al. 2015). Settings in Markov Chain Monte Carlo (MCMC) simulations were as follows: start from a random seed, 10 initialization attempts, five million pre-burnin iterations, followed by 50 million generations, and a sampling frequency of trees and parameters at 10,000. All BEAST analyses converged to the stationary distribution, with an effective sample size > 200 for all parameters. Altogether four independent MCMC runs were conducted for each alternative scenario, and the resulting tree files were merged in LogCombiner ver. 2.6.7. The final maximum clade credibility trees were summarized in TreeAnnotator ver. 2.6.7 (Bouckaert et al. 2014). A time-calibrated phylogenetic network was built from the unconstrained and constrained BEAST trees using the Julia library PhyloNetworks ver. 0.16.3 (Karimi et al. 2020).

Ancestral hosts of astome ciliates and their life histories were reconstructed for nodes of each alternative time-calibrated BEAST tree topology, using stochastic mapping and the SIMMAP function (Bollback 2006) as implemented in the R library phytools ver. 1.2-0 (Revell 2012). For settings and the flowchart of how the best fit evolutionary models were selected and the *Q* matrices and posterior probabilities of ancestral host groups and their life histories were computed, see Pecina et al. (2024). Finally, posterior probabilities were mapped onto the time-calibrated phylogenetic network using PhyloNetworks.

## Results

### Characterization of new sequences

The length and GC content of 18S varied from 1757 nt and 45.36% in *B. criodrili* through 1758 nt and 44.65% in *M. clavata* to 1763 nt and 44.64% in *H. secans*. ITS region-28S sequences were more variable in length and GC content than 18S: their length and GC content were 1337 nt and 47.12% in *B. criodrili*, 1357 nt and 43.70% in *M. clavata*, and 1328 nt and 45.86% in *H. secans*. 18S and ITS region-28S sequences were identical within species. The PCR amplified region of 16S included the C, 3′M, and 3′m domains. The length and GC content of this 16S fragment varied: 772 nt and 27.59% in *B. criodrili*, 950 nt and 36.53‒36.84% in *M. clavata*, and 995 nt and 35.38% in *H. secans*. No intraspecific variability occurred within *B. criodrili*, *H. secans*, or *M. clavata* populations, but the two *M. clavata* populations differed by 1.2%. A similar pattern occurred for the mitochondrial COI sequences; their length and GC content were 693 nt and 31.60‒34.46% in *B. criodrili*, 711 nt and 39.80‒40.08% in *M. clavata*, and 729 nt and 40.47% in *H. secans*. No intraspecific variability occurred within *H. secans*. Specimens of *B. criodrili* differed by a maximum of 0.2%. Similarly to 16S, no variability occurred within the two *M. clavata* populations, but COI sequences of both populations differed by 1.5%. The large interspecific and small intraspecific distances make the mitochondrial 16S and COI optimal DNA barcodes for astome ciliates. See Supplementary Table 3 for GenBank accession numbers.

### Phylogenetic analyses

**Fig. 2:**
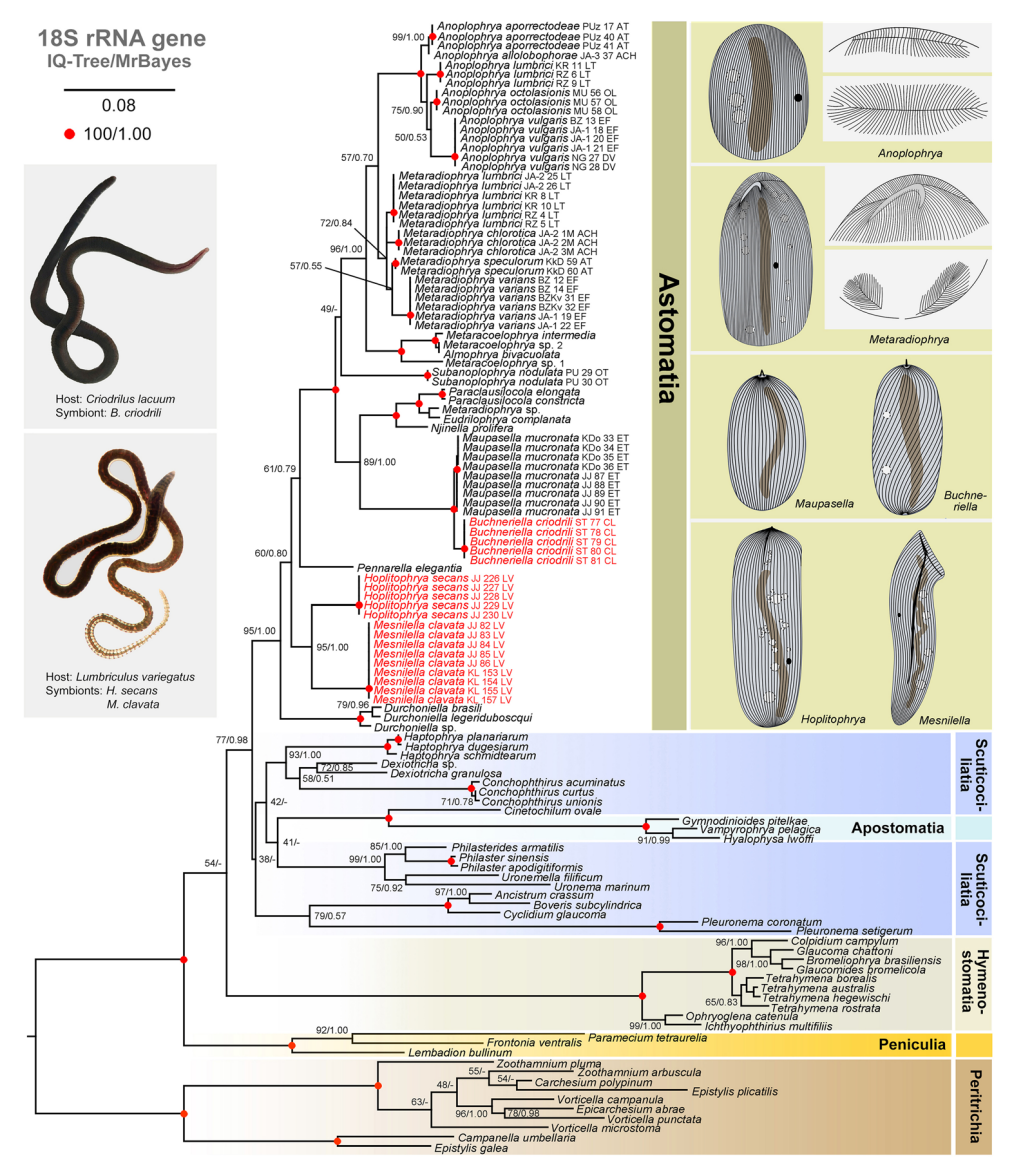
Phylogenetic tree based on the nuclear 18S rRNA gene sequences, showing the systematic positions of astome ciliates isolated from *Lumbriculus variegatus* and *Criodrilus lacuum*. The subclass Peritrichia was used to root the tree. Bootstrap values for maximum likelihood conducted in IQ-TREE and posterior probabilities for Bayesian inferences conducted in MrBayes were mapped onto the best scoring IQ-Tree. Sequences marked in red were obtained during this study. Fully statistically supported nodes are marked with red solid circles. Dash (‒) indicates a mismatch in tree topologies. The scale bar denotes eight substitutions per one hundred nucleotide positions

The monophyly of the subclass Astomatia was supported (95% ML bootstrap/1.00 Bayesian posterior probability [PP]) in the 18S trees (Fig. 2). However, relationships at the base of the astome clade were poorly resolved. Three *Durchoniella* species isolated from the marine polychaete *Cirriformia tentaculata* formed a fully supported monophylum that branched off first. Then, a strongly supported clade (95% ML/1.00 PP) comprising *H. secans* and *M. clavata*, both obtained from the freshwater *L. variegatus*, followed. *Pennarella elegantia*, isolated from the marine polychaete *Myxicola aesthetica*, never clustered with the polychaete-dwelling *Durchoniella* spp. but was placed in a sister-group position to all other astomes isolated from crassiclitellates (= true earthworms) (Fig. 2). Since the positions of the *Durchoniella*, *Pennarella*, and *Hoplitophrya‒Mesnilella* clades received a maximum ML bootstrap support of 61% and a posterior probability of 0.80, we considered their interrelationships to be unresolved. Therefore, we performed multiple tree topology tests (Table 1). The monophyletic origin of the analyzed polychaete-dwelling astomes, *Durchoniella* and *Pennarella*, and their basal position was only rejected by the RELL method. The monophyletic origin of the analyzed oligochaete-dwelling astomes, i.e., astomes isolated from lumbriculids (*Hoplitophrya‒Mesnilella* clade) and crassiclitellates (i.e., true earthworms), was not be refuted by any topology test (Table 1). The clade uniting crassiclitellate-dwelling astomes was fully supported by ML and Bayesian analyses. The position of *Subanoplophrya* was, however, unstable and weakly supported (Fig. 2). It either grouped with a clade comprising *Anoplophrya*, *Metaradiophrya*, and astomes isolated from African almid earthworms or with a clade comprising *Maupasella*, *Buchneriella*, and astomes isolated from African megascolecid earthworms. Any of these two scenarios could be excluded (Table 1). Adding further molecular markers (ITS region-28S and 16S) did not resolve the phylogenetic position of *Subanoplophrya*. The grouping of *Anoplophrya*, *Metaradiophrya*, and almid-dwelling astomes had strong support (96% ML/1.00 PP) in the 18S phylogenies (Fig. 2). The clustering of *Maupasella*, *Buchneriella*, and megascolecid-dwelling astomes was moderately to strongly supported (89% ML/1.00 PP) in the 18S trees. Phylogenetic interrelationships within these two clades were well resolved. However, the monophyly of *Metaradiophrya* and its kinship to *Anoplophrya* were poorly supported. Adding further molecular data (ITS region-28S, 16S, and COI) supported the monophyly of each genus and their sister-group relationship. However, the intra-generic branching pattern could not be well resolved, either in the six or seven molecular marker trees (Supplementary Fig. 1A, B). Topology tests could not reject the phylogenetic relationships suggested for *Anoplophrya* and *Metaradiophrya* in coalescent-based species trees from previous analyses (Table 1).

**Fig. 3:**
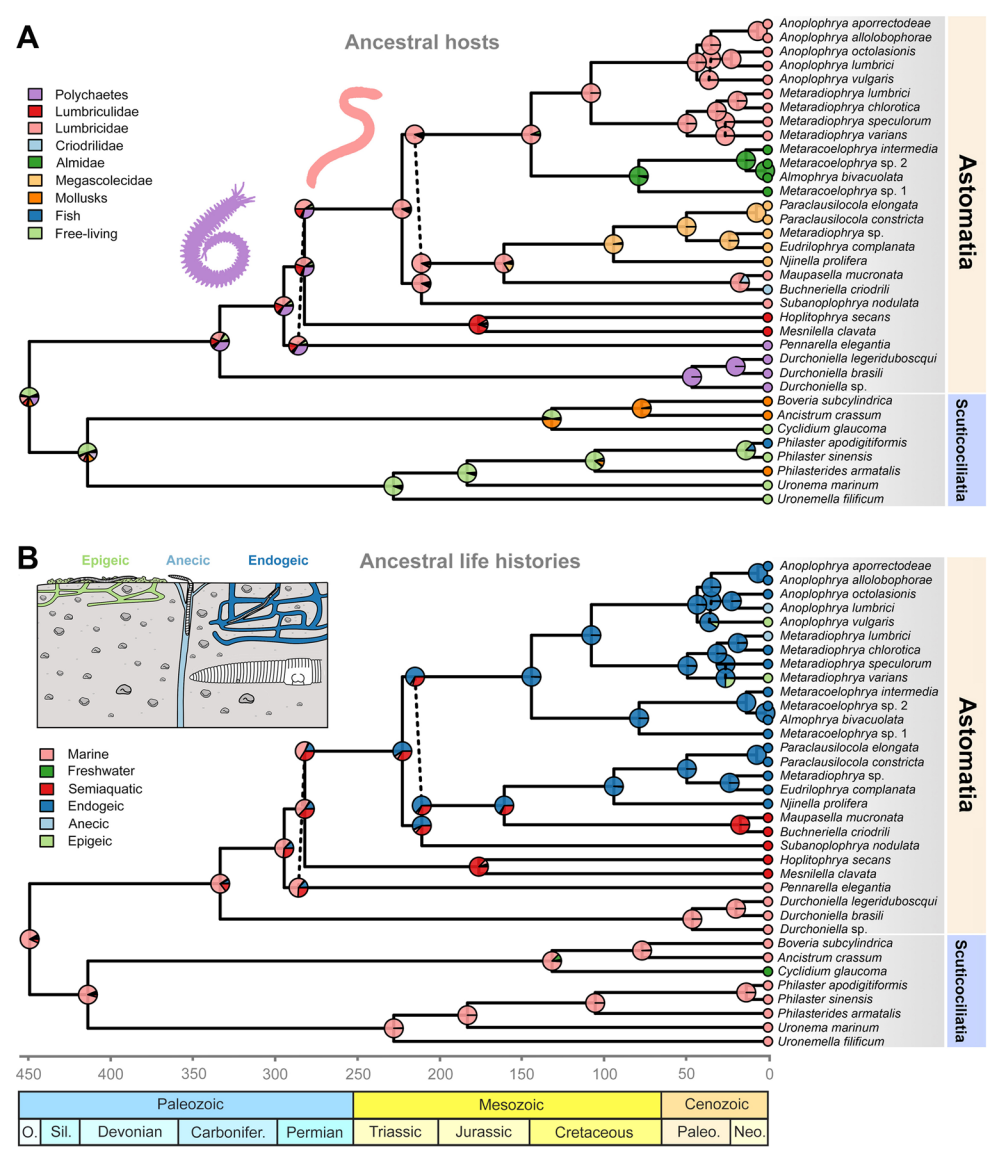
SIMMAP reconstruction of ancestral hosts (**A**) and life histories (**B**) of astome ciliates based on 10,000 stochastic character maps simulated under the equal rates (ER) model. Relative proportions of character states were mapped onto a phylogenetic network constructed from alternative time-calibrated maximum clade credibility trees built in BEAST. Dashed vertical lines indicate alternative evolutionary trajectories in the network. The time scale shows age in millions of years

SIMMAP analyses suggested that the last common ancestor of the subclass Astomatia colonized the digestive tract of polychaetes in the Paleozoic about 330 Ma (Fig. 3A). Astomes living in lumbriculids branched off during the early radiation of the subclass Astomatia. Lumbriculid-dwelling astomes of the genera *Hoplitophrya* and *Mesnilella* diverged in the Jurassic about 176 Ma. The radiation of astomes living in true earthworms took place in the Triassic about 223 Ma. According to SIMMAP reconstructions, the ancestral host of this diverse clade was a representative of the family Lumbricidae. In different time frames, astomes switched from Lumbricidae to Almidae, Megascolecidae, and Criodrilidae hosts. No backward transfers to lumbricids were detected (Fig. 3A).

The ancestral habitat of host annelids was likely the sea regardless of the phylogenetic position of the polychaete-dwelling *Pennarella* (Fig. 3B). SIMMAP analyses suggested at least three independent transfers to (semi)aquatic oligochaetes: (1) lumbriculids for *Hoplitophrya* and *Mesnilella*; (2) lumbricids for *Subanoplophrya*; and (3) criodrilids for *Buchneriella*. The ancestral host of the crassiclitellate-dwelling astome clade likely led an endogeic lifestyle. Mirroring the evolutionary history of life strategies of true earthworms, the transfer to epigeic and anecic earthworms happened from endogeic ancestors multiple times independently and in rather recent time frames, i.e., in the Eocene and Oligocene epochs about 35‒25 Ma (Fig. 3B).

### Ciliate taxonomy

- Phylum Ciliophora Doflein, 1901
- Class Oligohymenophorea de Puytorac et al., 1974
- Subclass Astomatia Schewiakoff, 1896
- Order Astomatida Schewiakoff, 1896
- Family Hoplitophryidae Cheissin, 1930
- Genus *Hoplitophrya* Stein, 1860 (type species: *Opalina secans* Stein, 1859)

## *Hoplitophrya secans* (Stein, 1859) Stein, 1861 (Figs. 4-6)

**Fig. 4:**
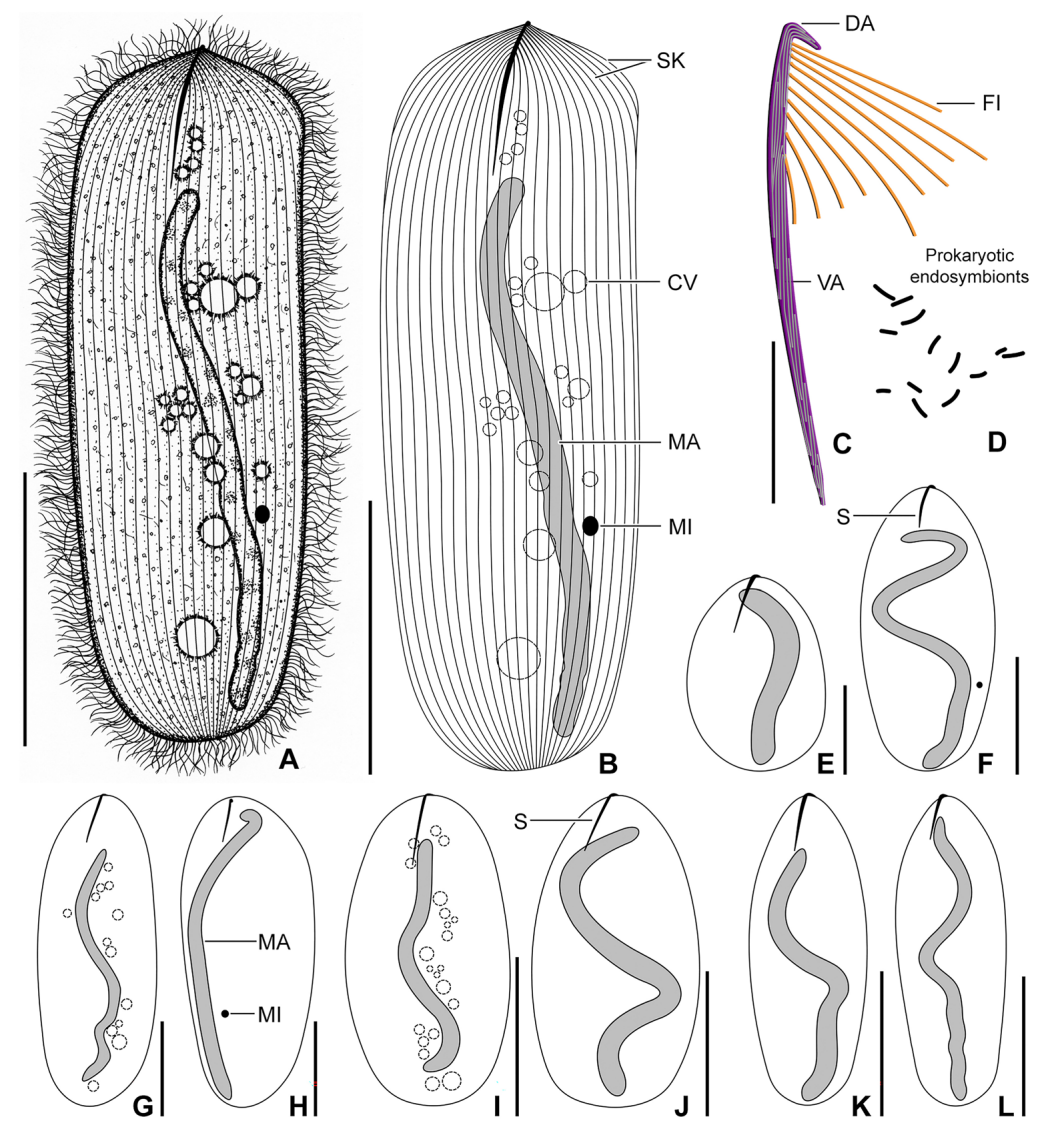
*Hoplitophrya secans* in vivo (**A**, **B**, **C**, **D**, **G**, **I**) and after protargol impregnation (**E**, **F**, **H**, **J**‒**L**), neotype population. **A** Ventral view of a representative specimen, showing the general body organization. **B** Semi-schematic diagram of the ventral side, showing the ciliary pattern, the nuclear and contractile vacuole apparatus, as well as the skeletal system. **C** Detail of the skeletal apparatus. **D** Prokaryotic cytoplasmic symbionts. **E**‒**L** Variability of body shape and size as well as of the nuclear and contractile vacuole apparatus. *CV* contractile vacuoles, *DA* dorsal spicule arm,* FI* fibers, *MA* macronucleus, *MI* micronucleus, *S* spicule, *SK* somatic kineties, *VA* ventral spicule arm. Scale bars: 20 μm (**C**), 50 μm (**E**), 100 μm (**A**, **B**, **F**‒**K**), 100 μm (**L**)

**Fig. 5:**
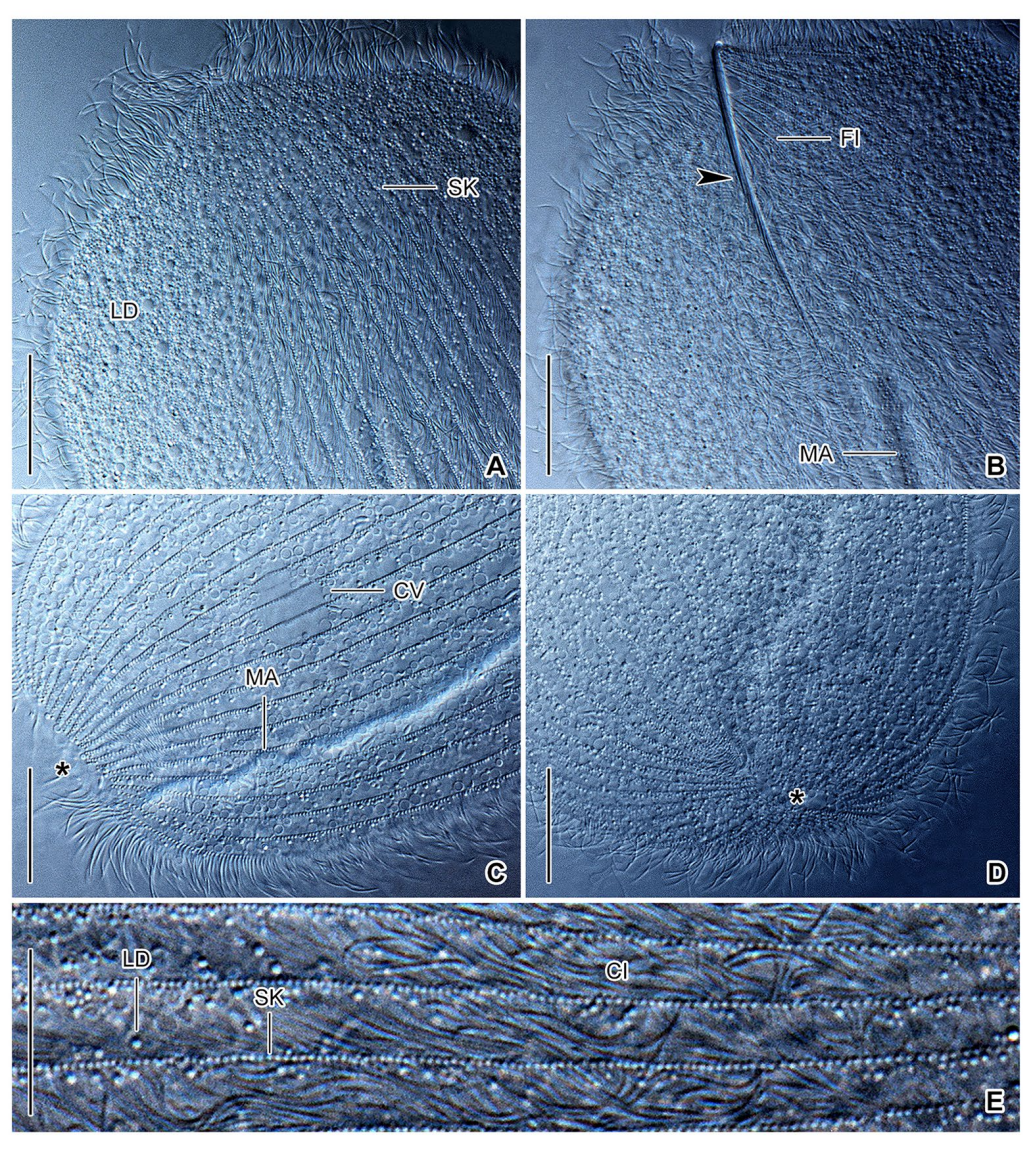
*Hoplitophrya secans* in vivo, neotype population (**A**‒**E**). **A**, **B** Details of the dorsal (**A**) and ventral (**B**) anterior body portion, showing the ciliary pattern and the skeletal system. Arrowhead denotes the ventral spicule arm. **C**, **D** Detail of the posterior body portion, showing the nuclear apparatus and the ciliary pattern. Note that all ciliary rows terminate slightly ahead of the posterior body pole, leaving a small blank field (asterisks). **E** Somatic cilia are very narrowly arranged, while somatic kineties are widely spaced. *CI* cilia, *CV* contractile vacuoles, *FI* fibers, *LD* lipid droplets, *MA* macronucleus, *SK* somatic kineties. Scale bars: 10 μm (**E**), 20 μm (**A**‒**D**)

**Fig. 6:**
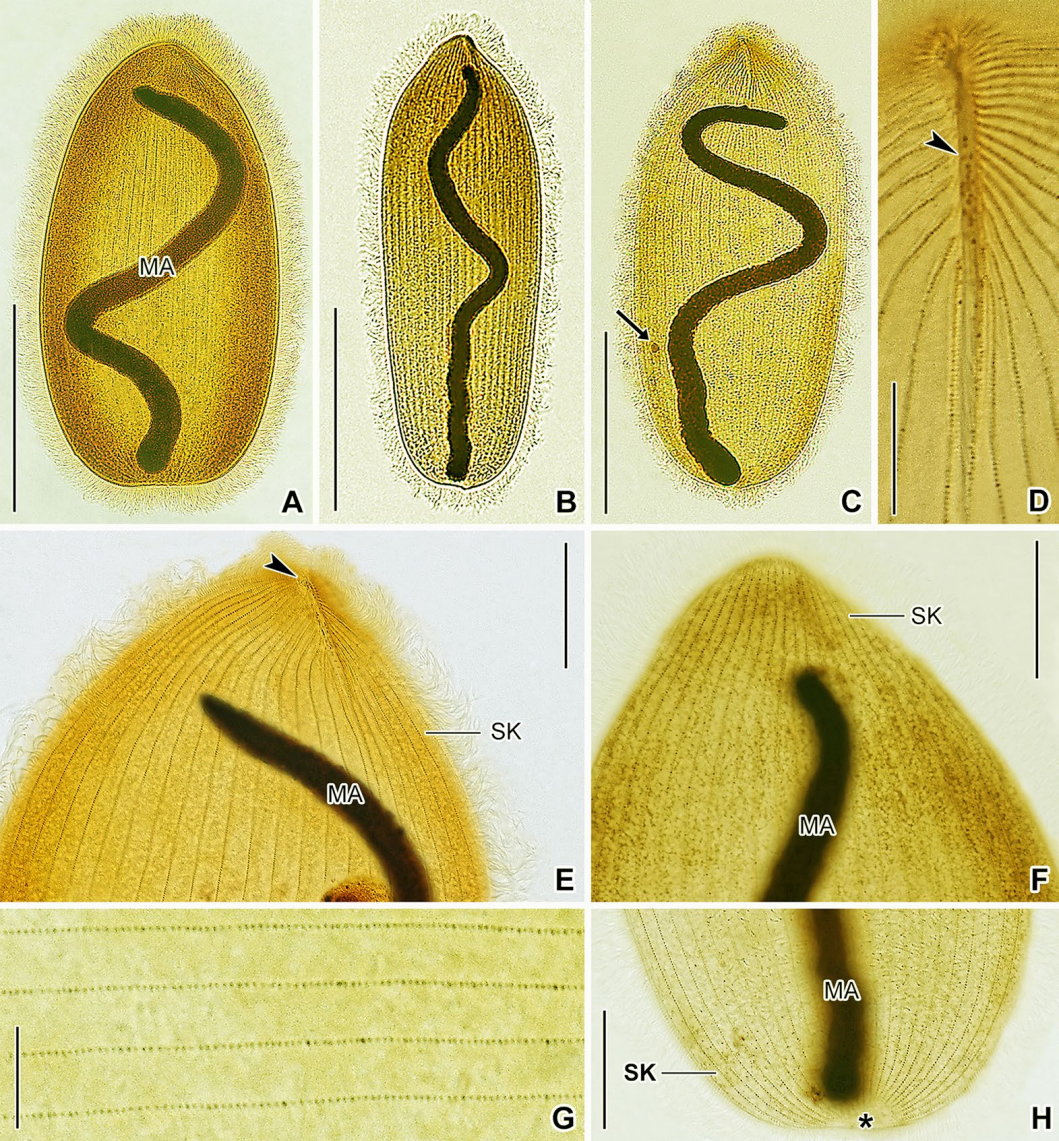
*Hoplitophrya secans* after protargol impregnation, neotype population (**A**‒**H**). **A**‒**C** Variability of body shape and size as well as of the nuclear apparatus. Arrow denotes the micronucleus. **D** Detail of the ciliary pattern around the ventral spicule arm (arrowhead). **E**, **F** Details of the ventral (**E**) and dorsal (**F**) anterior body portion, showing the ciliary pattern. Arrowhead denotes the ventral spicule arm. **G** Somatic kineties are widely spaced and composed of very narrowly arranged basal bodies. **H** Detail of the posterior body portion, showing the ciliary pattern. Ciliary rows end slightly ahead of the posterior body pole, leaving a small blank field (asterisks). *MA* macronucleus, *SK* somatic kineties. Scale bars: 10 μm (**D**, **G**), 30 μm (**E**, **F**, **H**), 100 μm (**A**, **C**), 200 μm (**B**)

### Synonymy

Heidenreich (1935) and de Puytorac (1957, 1960, 1972) reviewed the extensive synonymy history of *H. secans*. We assessed their synonymy lists and surveyed the old literature. The following records could be assigned to *H. secans*:

1859 *Opalina secans* Stein, Abhandlungen der Königlichen böhmischen Gesellschaft der Wissenschaften, 5. Folge, Band 10, years 1857‒1859: 36 (brief description without figure, type hosts: *Saenuris variegata* and *Enchytraeus vermicularis*)
1860 *Opalina secans* St. ‒ Stein, Sitzungsberichte der Königlichen böhmischen Gesellschaft der Wissenschaften in Prag year 1860: 56 (listing the species, without figure)
1861 *Hoplitophrya secans* ‒ Stein, Sitzungsberichte der Königlichen böhmischen Gesellschaft der Wissenschaften in Prag year 1861: 88 (combining author, correction of type host name from *Saenuris variegata* to *Lumbriculus variegatus*)
1862 *Opalina lumbriculi* mihi ‒ Buchholz, Schriften der Königlichen physikalisch-ökonomischen Gesellschaft zu Königsberg year 1863: 130 (size 580 × 21 μm; host: not provided, very likely *Lumbriculus* as suggested by the species epithet)
1881 *Hoplitophrya secans* Stein ‒ Kent, Manual of Infusoria II: 572 (brief description without figure; hosts: *Lumbriculus variegatus* and *Enchytraeus vermicularis*)
1926 *Hoplitophrya lumbriculi* Kijenskij, Věstník Královské české společnosti nauk, Třída matematicko-přírodovědecká year 1925 (I): 8, Obr. 4, 6, Tab. I obr. 2 (size 225‒240 × 20‒30 μm, spicule 45 μm long, 10‒13 contractile vacuoles; host: *Lumbriculus variegatus*)
1929 *Protoradiophrya armata* Rossolimo and Perzewa, Archiv für Protistenkunde 67: 247, Figs. 11‒17 (size 285‒665 × 24‒32 μm, spicule 40‒45 μm long, 25‒30 contractile vacuoles; host: *Lumbriculus variegatus*)
1935 *Hoplitophrya secans* Stein ‒ Heidenreich, Archiv für Protistenkunde 84: 326, Fig. 1 (taxonomic revision, illustration showing the skeletal apparatus)
1954 *Hoplitophrya secans* Stein, 1861 ‒ Meier, Archiv für Protistenkunde 100: 217 (notes on a German population, 280‒900 × 18‒45 μm in size, without figure, observed together with *M. clavata*; host: *Lumbriculus variegatus*)
1957 *Hoplitophrya secans* (Stein 1859) ‒ de Puytorac, Archives de zoologie expérimentale et générale 94: 104 (brief synonymy list)
1960 *Hoplitophrya secans* ‒ de Puytorac, Journal of Protozoology 7: 279, Fig. 2 (microphotograph of the anterior body region showing the fibrillar spicule)
1972 *Hoplitophrya secans* (Stein 1859) Stein, 1861 ‒ de Puytorac, Protistologica 8: 10, Fig. 1, 2, Tab. 2 Fig. 14 (taxonomic revision, microphotograph and illustration of the ciliary pattern in the anterior body region, 240‒500 × 20‒60 μm in size, spicule 60‒70 μm long, 44 ciliary rows, 15‒30 contractile vacuoles; host: *Lumbriculus variegatus*)

### Misidentifications

Although Heidenreich (1935) and de Puytorac (1972) assigned the following records from Lankester (1870), Warpachowsky (1886), Bütschli (1889), Schewiakoff (1896), and Cépède (1910) to *H. secans*, their conspecificity could be excluded due to morphological differences and/or very different host organisms. Therefore, the following records should not be attributed to *H. secans*:

1870 *Opalina naidos* Dujardin ‒ Lankester, Quarterly Journal of Microscopical Science 10: 143, Pl. IX (differs from *H. secans* by contractile vacuoles arranged in a row, the thick macronucleus extending along most of the cell length, and by the lack of fibrillar spicule; hosts: *Nais serpentina* and *Lumbriculus* sp.; Cépède (1910) suggested transfer of *O. naidos* to *Anoplophrya*)
1886 *Opalina spiculata* Warpachowsky, Bulletin de l’Académie impériale des sciences de Saint-Pétersbourg 30: 513 (differs from *H. secans* by the long fibrillar spicule occupying ~ 66% of the body length; host: not mentioned)
1889 *Hoplitophrya* (?) *secans* St. ‒ Bütschli, Bronn’s Klassen und Ordnungen des Thierreichs: 1717, Taf. 65, Fig. 4a‒b (likely a misidentification because fission anisotomic with catenulation, i.e., with chain formation; host: *Nais*)
1896 *Hoplitophrya secans* Stein ‒ Schewiakoff, Mémoires de l’Académie impériale des sciences de Saint-Pétersbourg 4: 385, Tab. 6 Fig. 147 (Schewiakoff 1896 considered his species to be conspecific with *Opalina spiculata* sensu Warpachowsky 1886; hosts: *Lumbricus terrestris*, *Lumbricus variegatus*, and *Enchytraeus vermicularis*)
1910 *Mesnillella secans* Stein ‒ Cépède, Archives de zoologie expérimentale et générale 5: 550, Fig. XXXII (likely a misidentification because fibrillar spicule extends behind the mid-body; hosts: *Lumbricus terrestris* and *Lumbricus variegatus*)

### Neotypification

We neotypify *H. secans* with a Slovak population from the Jurské jazierko pond for the following objections: (1) the original description is incomplete, lacks figures, and the type locality is not known (see Art. 75.3.1 of the ICZN 1999); (2) there are several similar species that might be confused with the species to be neotypified (see above), and characters differentiating *H. secans* from congeners are provided in ‘Improved diagnosis’ below (see Art. 75.3.2); (3) the neotype is properly labeled to ensure its recognition (see Art. 75.3.3); (4) no type material is available from the species described by Stein (1859) (see Art. 75.3.4); (5) the neotype matches the species considered to be *H. secans* in the authoritative revision of de Puytorac (1972) (see Art. 75.3.5); (6) the neotype is from the same biogeographic region (Palearctic) and type host (*L. variegatus*) (see Art. 75.3.6 and Foissner 2002); and (7) genomic DNA, as well as genetic data from the nuclear rDNA operon and two mitochondrial genes are available for the neotype and have been deposited in a recognized scientific institution (see Art. 75.3.7 and ‘Type material’ below) and in the GenBank database (https://www.ncbi.nlm.nih.gov/nucleotide/).

### Improved diagnosis (based on neotype population)

Body size 200‒350 × 100‒130 μm in vivo. Shape broadly to narrowly elliptical to ovate. Fibrillar spicule extends along midline in anterior body fifth, composed of two unevenly long arms: ventral arm 41‒58 μm long, dorsal arm inconspicuous, hook-like, and only 6.0‒7.5 μm long; left side of ventral spicule arm associated with approximately 10 fibers running underneath ciliary rows. Macronucleus rod-like to filiform and tortuous, micronucleus globular and situated in posterior body half close to macronucleus. About 22 contractile vacuoles form an irregular and wide stripe along macronucleus. About 20‒25 meridional ciliary rows both on ventral and dorsal body sides, ventral rows gradually commence right and left of long spicule arm while dorsal rows start at anterior body pole, all ciliary rows terminate slightly ahead of posterior body pole leaving a small blank field.

### Type locality

Not provided in the original description. The neotype is from the Jurské jazierko pond situated in an urban oak-hornbeam forest, district of the village of Svätý Jur, Malé Karpaty Mts., Slovakia, 48°15′28.0″N 17°09′14.6″E. According to Article 76.3 of the ICZN (1999), the place of origin of the neotype becomes the type locality.

### Type host

Stein (1859) mentioned that *H. secans* was found in the intestine of *Saenuris variegata* and *Enchytraeus vermicularis*. Later, Stein (1861) corrected the name of the type host from *Saenuris variegata* (Hoffmeister, 1843) to *Lumbriculus variegatus*. ND1 and COI sequences of the *L. variegatus* individual (JJ 58) carrying the neotype specimen of *H. secans* have been deposited in GenBank under the following accession numbers: PQ247164 and PQ243270, respectively.

### Type material

No material is available from the type population. A DNA sample of the neotype specimen (JJ 226 LV) has been deposited in the Natural History Museum, Vajanského nábrežie 2, 810 06 Bratislava, Slovakia (ID Collection Code 01427561). The 18S rRNA gene, ITS1-5.8S-ITS2-28S rRNA gene, 16S rRNA gene, and cytochrome *c* oxidase subunit I sequences of the neotype specimen have been deposited in GenBank under the following accession nos. PQ238882, PQ240648, PQ249009, and PQ247145, respectively.

### Etymology

The species epithet *secāns* [m, f, n] (cutting, cleaving, dividing) is a participle in the nominative singular of the Latin verb *secō* (cut, cleave) (Article 11.9.1.1 of the ICZN 1999). The species-group name very likely refers to the asymmetric (anisotomic) cell division, causing the opisthe to be much smaller than the proter.

### Description of neotype population

Body size ~ 200‒350 × 100‒130 μm in vivo and 216‒450 × 110‒160 μm after protargol impregnation, length:width ratio 2.0‒2.8:1 both in vivo and in protargol preparations; dorso-ventrally flattened, not contractile. Body outline broadly to narrowly elliptical to ovate, often with anterolateral shoulders providing an angular appearance to living cells; cell apex appears spiny in vivo at low magnifications due to fibrillar spicule extending from ventral side onto dorsal one; anterior end usually slightly more narrowly rounded than posterior end; posterior end narrowly to broadly rounded, sometimes bluntly truncated (Figs. 4A, B, E–L, 5A‒D, 6A‒C, E, F, H).

Fibrillar spicule extends along midline of cell in anterior body fifth, composed of two unevenly long arms: ventral arm distinctly longer, i.e., about 41‒58 μm long, while dorsal arm inconspicuous, hook-like, and only 6.0‒7.5 μm long; left side of ventral spicule arm associated with ~ 10 fibers running underneath ciliary rows and well recognizable with differential interference contrast optics (Figs. 4A‒C, E‒L, 5B).

Nuclear apparatus begins close to anterior body end or slightly posterior to rear end of ventral spicule arm, extends almost to posterior body end both in vivo and in protargol preparations. Macronucleus rod-like to filiform and tortuous, ~ 210‒300 μm long in vivo when uncoiled, 10‒15 μm wide in vivo while 8‒11 μm after protargol impregnation; nucleoli small and globular to ellipsoidal, evenly distributed over macronucleus. Micronucleus 6 μm in diameter after protargol impregnation, close but not attached to macronucleus, situated in posterior body half, not recognized in vivo and only rarely observed in protargol preparations (Figs. 4A, B, E–L, 5C, 6A‒C). As many as 22 contractile vacuoles forming an irregular and comparatively wide stripe along macronucleus; excretory pores observed neither in vivo nor after protargol impregnation (Figs. 4A, B, G, I, 5C). Cortex flexible; not, or only slightly, furrowed by ciliary rows; no specific granules recognizable in vivo. Cytoplasm colorless, hyaline, contains lipid droplets and rod-like prokaryotes about 3.5 μm long in vivo, prokaryotic endosymbionts weakly impregnate with the protargol method used (Figs. 4D, 5A‒E).

Somatic ciliature holotrichous and composed of monokinetids. Somatic cilia about 11 μm long in vivo, very narrowly spaced and arranged in 20‒25 meridional rows both on ventral and dorsal body sides. Individual ciliary rows widely spaced, i.e., interkinetal distance ~ 4.2 μm after protargol impregnation. Ventral ciliary rows gradually commence right and left of long spicule arm, while dorsal ciliary rows start at anterior body pole (Figs. 4B, 5A, C, E, 6D–G). All ciliary rows terminate slightly ahead of posterior body pole, leaving an inconspicuous blank field recognizable both in vivo and after protargol impregnation (Figs. 5D, 6H, asterisk).

Genus *Mesnilella* Cépède, 1910 (type species: *Leucophrys clavata* Leidy, 1855)

## *Mesnilella clavata* (Leidy, 1855) Cépède, 1910 (Figs. 7-9)

**Fig. 7:**
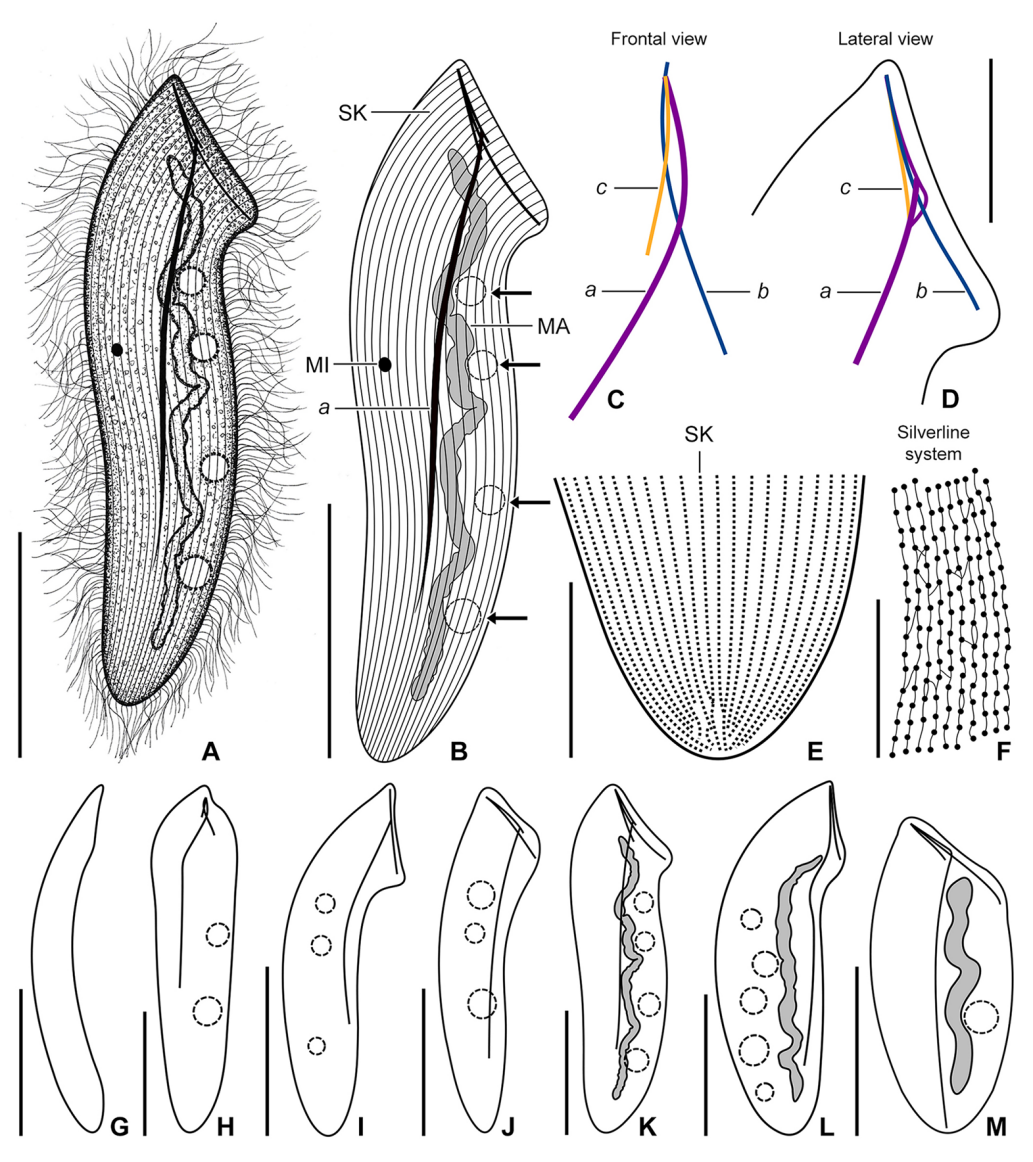
*Mesnilella clavata* in vivo (**A**‒**D**, **G**‒**M**), after protargol (**E**) and silver nitrate (**F**) impregnation, neotype population. **A** Ventral view of a representative specimen, showing the general body organization. **B** Semi-schematic diagram, showing the ciliary pattern, the nuclear and contractile vacuole apparatus (arrows), as well as the skeletal system. **C**, **D** Details of the skeletal apparatus. **E** Detail of the posterior body portion, showing the ciliary pattern. **F** Silverline system. **G**‒**M** Variability of body shape and size as well as of the nuclear and contractile vacuole apparatus. *a‒c* fibers *a‒c*, *MA* macronucleus, *MI* micronucleus, *SK* somatic kineties. Scale bars: 10 μm (**F**), 20 μm (**D**, **E**), 40 μm (**M**), 50 μm (**A**, **B**, **G**‒**L**)

**Fig. 8:**
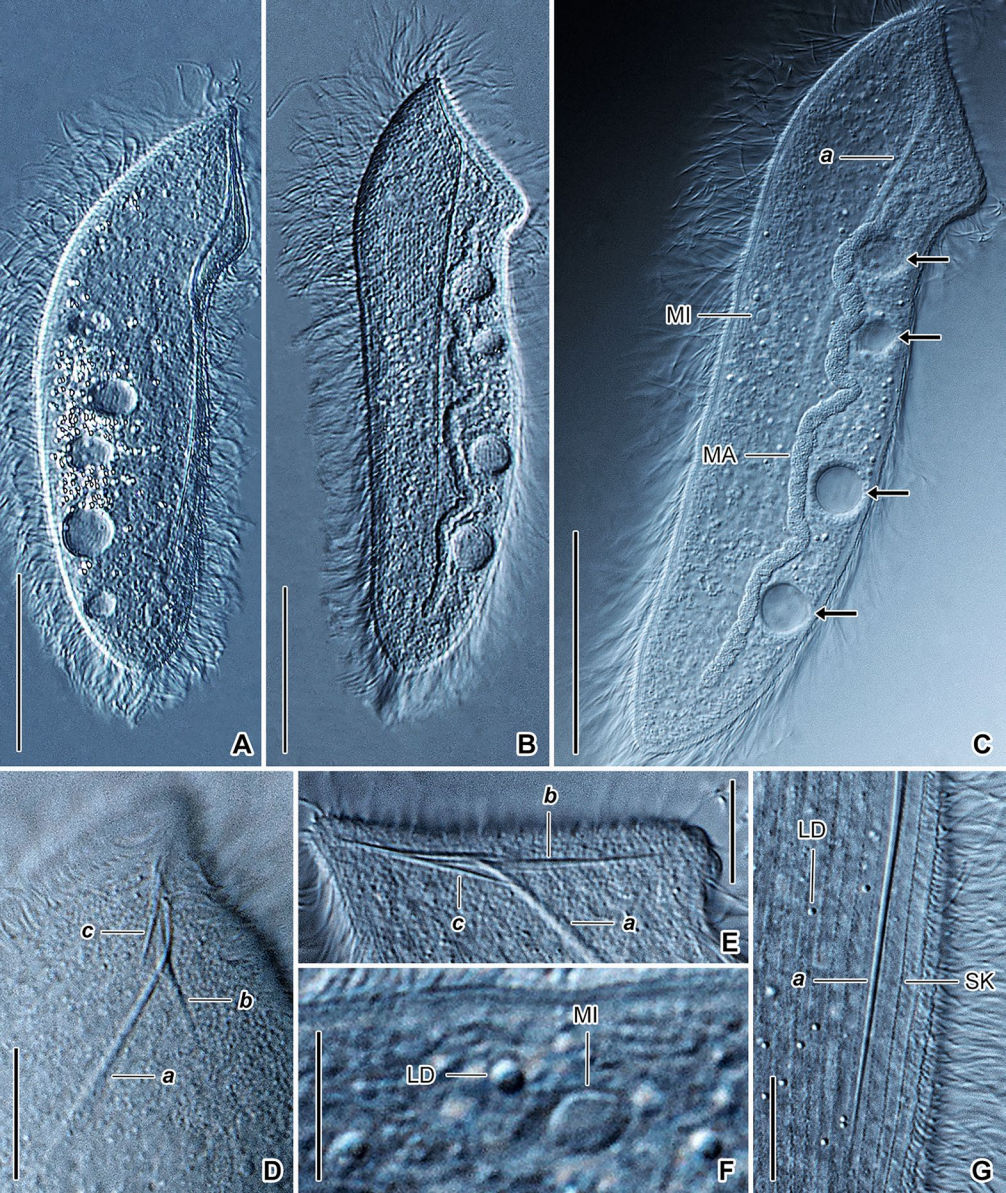
*Mesnilella clavata* in vivo, neotype population (**A**‒**G**). **A**‒**C** Variability of body shape and size as well as of the nuclear apparatus. Arrows in (**C**) denote contractile vacuoles. **D**, **E** Details of the skeletal apparatus. **F** Detail showing the micronucleus, lipid droplets, and innumerable granules scattered throughout the cytoplasm. **G** Detail showing the posterior portion of fiber *a*. *a‒c* fibers *a‒c*, *LD* lipid droplets, *MA* macronucleus, *MI* micronucleus, *SK* somatic kineties. Scale bars: 5 μm (**F**), 10 μm (**D**, **E**, **G**), 40 μm (**A**‒**C**)

**Fig. 9:**
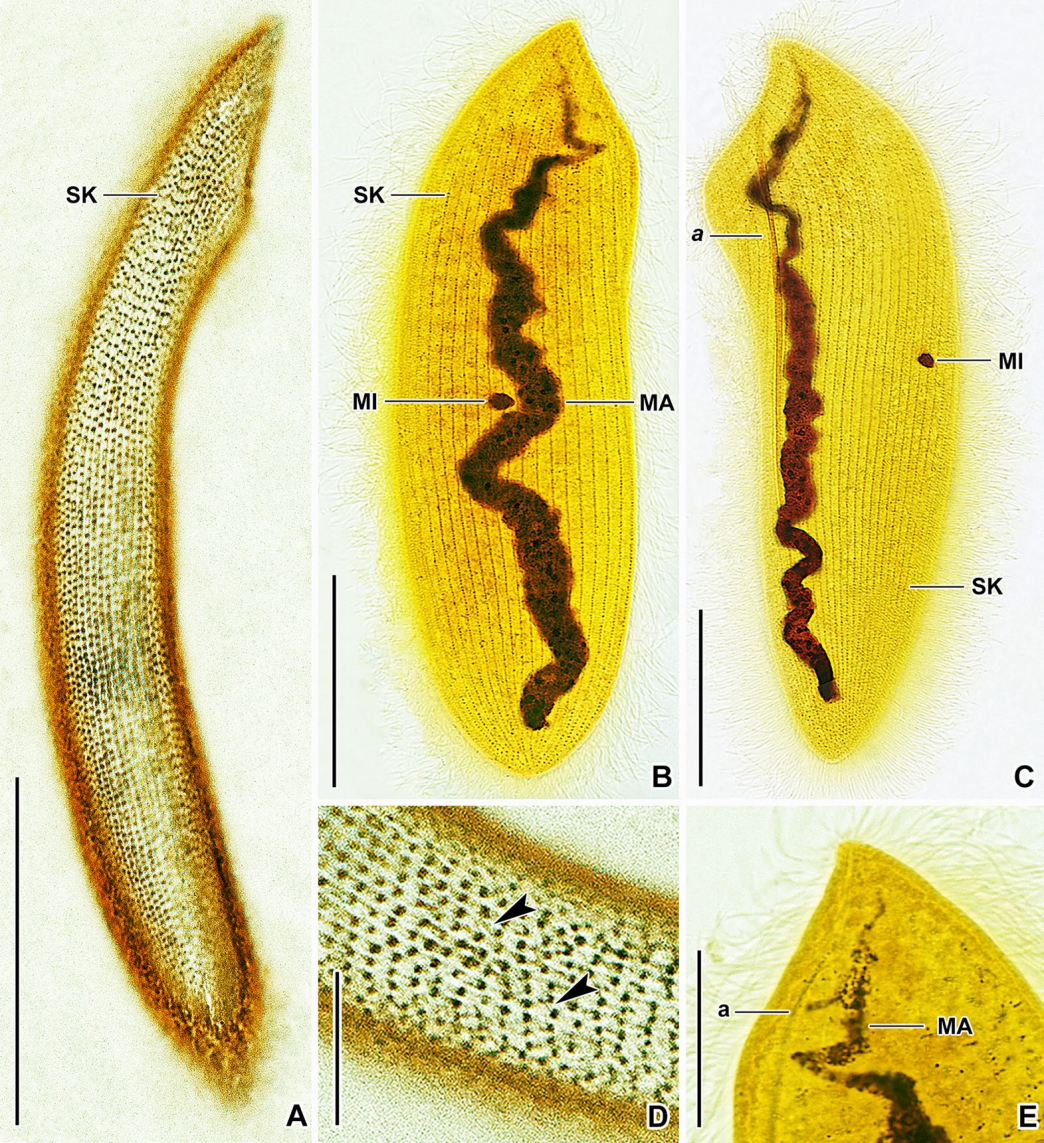
*Mesnilella clavata* after silver nitrate (**A**, **D**) and protargol (**B**, **C**, **E**) impregnation, neotype population. **A** Silverline system of the right body side. **B**, **C** Right (B) and left (C) view of representative specimens, showing the ciliary pattern and nuclear apparatus. **D** Detail of the silverline system. Arrowheads mark short silverlines connecting every fourth to fifteenth basal body of adjacent somatic kineties. **E** Detail of the anterior portion of the macronucleus. Note that the macronucleus narrows and fades in its anterior portion. *a* fiber *a*, *MA* macronucleus, *MI* micronucleus, *SK* somatic kineties. Scale bars: 10 μm (**D**), 20 μm (**E**), 40 μm (**A**‒**C**)

### Synonymy

Heidenreich (1935) and de Puytorac (1957, 1972) assembled the synonymy history of *M. clavata*. We critically reviewed and updated their synonymy lists:

1855 *Leucophrys clavata* Leidy, Journal of the Academy of Natural Sciences of Philadelphia 3: 144 (without description and figure, type locality: Rhode Island and New Jersey; host: *Lumbriculus tenuis*)
1861 *Hoplitophrya securiformis* Stein, Sitzungsberichte der Königlichen böhmischen Gesellschaft der Wissenschaften in Prag: 89 (brief description without figure; host: *Lumbriculus variegatus*)
1881 *Anoplophrya clavata* Leidy ‒ Kent, Manual of Infusoria II: 566, Pl. XXVI Fig. 21 (brief description with figure; host: *Lumbriculus limosus* (Leidy, 1850) [= *Lumbriculus variegatus* (Müller, 1774)])
1881 *Hoplitophrya securiformis* Stein ‒ Kent, Manual of Infusoria II: 573 (brief description without figure; host: *Lumbriculus variegatus*)
1889 *Hoplitophrya clavata* Leidy ‒ Bütschli, Bronn’s Klassen und Ordnungen des Thierreichs: 1717, Taf. 65, Fig. 4c (listed, with figure; host: not mentioned)
1896 *Hoplitophrya clavata* Leidy sp. ‒ Schewiakoff, Mémoires de l’Académie impériale des sciences de Saint-Pétersbourg 4: 386 (description without figure; host: *Lumbriculus variegatus*)
1910 *Anoplophrya* (?) *clavata* Leidy ‒ Cépède, Archives de zoologie expérimentale et générale 5: 536, Fig. XXIV (brief description with figure; host: *Lumbriculus limosus* (Leidy, 1850) [= *Lumbriculus variegatus* (Müller, 1774)])
1910 *Mesnilella clavata* Leidy ‒ Cépède, Archives de zoologie expérimentale et générale 5: 551, Fig. XXXIII (combining author, brief description with figure; host: *Lumbriculus variegatus*)
1912 *Hoplitophrya clavata* (Leidy sp. Bütschli) ‒ Schuster, Spisů poctěných jubilejní cenou Královské české společnosti náuk v Praze 21: 19 (listed, without figure; host: *Lumbriculus variegatus*)
1922 *Mesnilella clavata* (Leidy) Cépède (1910) ‒ Penard, Études Infusoires: 11 (detailed description with figure, description of cysts; host: *Lumbriculus*)
1924 *Mesnilella clavata* (Leidy) ‒ Mackinnon and Adam, Quarterly Journal of Microscopical Science 68: 224 (detailed description with figure; host: *Lumbriculus variegatus*)
1926 *Mesnilella clavata* (Leidy) ‒ Kijenskij, Věstník Královské české společnosti nauk, Třída mathematicko-přírodovědecká year 1925 (I): 2, Obr. 1, Tab. 1 obr. 1 (detailed description with figures; host: *Lumbriculus variegatus*)
1930 *Anoplophrya clavata* Leidy ‒ Cheissin, Archiv für Protistenkunde 70: 608 (only listed, without figure)
1930 *Mesnililla clavata* Leidy ‒ Cheissin, Archiv für Protistenkunde 70: 610 (only listed, without figure, incorrect subsequent spelling of the genus name and therefore unavailable according to Articles 33.3 and 33.5 of the ICZN 1999)
1935 *Mesnilella clavata* Leidy ‒ Heidenreich, Archiv für Protistenkunde 84: 341 (revision, description without figure)
1954 *Mesnilella clavata* (Leidy 1855) ‒ Meier, Archiv für Protistenkunde 100: 230 (brief description of a German population, without figure, observed together with *H. secans*; host: *Lumbriculus variegatus*)
1957 *Mesnilella clavata* (Leidy 1855) ‒ de Puytorac, Archives de zoologie expérimentale et générale 94: 104 (brief synonymy list)
1965 *Mesnilella clavata* ‒ Kaczanowski, Acta Protozoologica 3: 227, Fig. 2D (only figure provided)
1972 *Mesnilella clavata* (Leidy, 1855) Cépède, 1910 ‒ de Puytorac, Protistologica 8: 12, Fig. 4, Tab. II Fig. 23 (revision, figure showing the anterior body portion; host: *Lumbriculus variegatus*)

### Neotypification

We neotypify *M. clavata* with a Slovak population from the Jurské jazierko pond for the following reasons: (1) the species lacks original description and figures (see Art. 75.3.1 of the ICZN 1999); (2) characters differentiating *M. clavata* from congeners are provided in ‘Improved diagnosis’ below (see Art. 75.3.2); (3) the neotype is properly labeled to ensure its recognition (see Art. 75.3.3); (4) it is generally known that no type material is available from Leidy (1855) (see Art. 75.3.4); (5) the neotype matches very well the species considered to be *M. clavata* in the authoritative revisions of Cépède (1910), Penard (1922), Kijenskij (1926), and de Puytorac (1972) (see Art. 75.3.5); (6) the neotype is from the same biogeographic region (Holoarctic) and type host oligochaete (Stein 1861 stated that “*Lumbriculus tenuis*” mentioned by Leidy 1855 is, in fact, *L. variegatus*) (see Art. 75.3.6 and Foissner 2002); and (7) genomic DNA, as well as genetic data from the nuclear rDNA operon and two mitochondrial genes are available for the neotype and have been deposited in a recognized scientific institution (see Art. 75.3.7 and ‘Type material’ below) and in the GenBank database (https://www.ncbi.nlm.nih.gov/nucleotide/).

### Improved diagnosis (based on neotype population)

Body size 125‒150 × 35‒45 μm in vivo. Shape clavate to elongate elliptical, with anterior body end slanted, posterior end narrowly rounded, and both body margins slightly sigmoidal. Skeletal apparatus composed of three intertwined and unequally long fibers: fiber *a* ~ 100 μm long, begins at cell apex, runs underneath anterior body margin and soon turns posteriorly to extend along midline of cell; fiber *b* ~ 30 μm long, runs underneath truncated anterior body margin to terminate before reaching left body margin; fiber *c* only 18 μm long in vivo, runs slightly obliquely with respect to fibers *a* and *b* to terminate about in mid-portion of anterior body margin. Macronucleus filiform and tortuous, micronucleus broadly ellipsoidal to broadly fusiform and usually situated far away from macronucleus close to mid-portion of right body margin. Usually 4‒6 contractile vacuoles arranged in a single row extending along macronucleus. About 20‒23 meridional ciliary rows both on ventral and dorsal body sides.

### Type locality

Leidy (1855) mentioned three sites where he recorded *Leucophrys clavata*: Point Judith (Rhode Island), Absecom Beach (Atlantic City), and Beesley’s Point (New Jersey). The neotype is from the Jurské jazierko pond situated in an urban oak-hornbeam forest, district of the village of Svätý Jur, Malé Karpaty Mts., Slovakia, 48°15′28.0″N 17°09′14.6″E. According to Article 76.3 of the ICZN (1999), the place of origin of the neotype becomes the type locality.

### Type host

Leidy (1855) isolated *Leucophrys clavata* from the visceral cavity (presumably the digestive tract) of *Lumbriculus tenuis*. Later, Stein (1861) corrected the host name to *L. variegatus*. ND1 and COI sequences of the *L. variegatus* individual (JJ 37) carrying the neotype specimen of *M. clavata* have been deposited in GenBank under the following accession numbers: PQ247165 and PQ243271, respectively.

### Type material

No material is available from the type population. A DNA sample of the neotype specimen (JJ 82 LV) has been deposited in the Natural History Museum, Vajanského nábrežie 2, 810 06 Bratislava, Slovakia (ID Collection Code 01427562). The 18S rRNA gene, ITS1-5.8S-ITS2-28S rRNA gene, 16S rRNA gene, and cytochrome *c* oxidase subunit I sequences of the neotype specimen have been deposited in GenBank under the following accession nos. PQ238887, PQ240653, PQ249014, and PQ247150, respectively.

### Etymology

The species epithet is derived from the Latin noun *clāv*·*a*, -*ae* [f] (club, cudgel) and used as an adjective in the nominative singular (Article 11.9.1.1 of ICZN 1999). The species-group name alludes to the club-shaped (clavate) body of the species.

### Description

*Mesnilella clavata* was recorded in *L. variegatus* collected at two locations: Jurské jazierko pond, district of the village of Svätý Jur, Malé Karpaty Mts. (48°15′28.0″N 17°09′14.6″E) and Kráľovská lúka, riparian zone of the River Danube (47°52′59.5″N 17°31′02.8″E). The conspecificity of both populations was confirmed by three nuclear rRNA genes (18S, 5.8S, and 28S), their spacers (ITS1 and ITS2), as well as by the barcoding mitochondrial COI and 16S rRNA genes. Since both populations match very well also morphologically, the description combines all observations.

Body size about 125‒150 × 35‒45 μm in vivo and 130‒160 × 40‒45 μm after protargol impregnation, length:width ratio 3.2‒3.6:1 both in vivo and in protargol preparations; dorso-ventrally flattened, not contractile. Shape clavate to elongate elliptical, with both body margins slightly sigmoidal. Anterior body end straight, 30‒35 μm long in vivo, and slanted by about 45° providing the living cells with a *Spathidium*-like appearance. Posterior body end usually narrowly rounded, rarely broadly rounded in some protargol-impregnated cells (Figs. 7A, B, G–M, 8A‒C, M, 9A‒C).

Skeletal apparatus composed of three intertwined and unequally long fibers originating at cell apex (i.e., at place where anterior margin meets right cell margin). Fiber *a* most conspicuous because robust and very long (ca. 100 μm); begins at cell apex, runs underneath anterior body margin, soon turns posteriorly to extend along midline of cell; occupies about 70% of body length; faintly impregnates with the protargol method used. Fiber *b* about 30 μm long in vivo; runs underneath truncated anterior body margin to terminate before reaching left body margin, i.e., occupies ca. 90% of anterior margin length; not recognizable after protargol impregnation. Fiber *c* shortest, i.e., only 18 μm long in vivo; runs slightly obliquely with respect to fibers *a* and *b* to terminate about in mid-portion of anterior body margin; not recognizable after protargol impregnation (Figs. 7A–D, H‒M, 8A‒E, G, 9C, E).

Nuclear apparatus begins about 12 μm apart from cell apex and terminates about 15 μm ahead of posterior body end, occupying ca. 80% of body length. Macronucleus filiform and tortuous, with anterior portion sometimes narrowing and fading in protargol preparations (very likely opisthe post-dividers), about 90‒100 μm long in vivo when uncoiled, 3‒5 μm wide in vivo and 3‒7 μm after protargol impregnation; nucleoli small and more or less globular to ellipsoidal, evenly distributed over macronucleus. Micronucleus broadly ellipsoidal to broadly fusiform, 3.5‒4.5 μm in largest diameter after protargol impregnation, typically situated conspicuously far away from macronucleus, viz., close and slightly above mid-portion of right body margin, only rarely close but not attached to macronucleus (Figs. 7A, B, K–M, 8B, C, F, 9B, C, E). Usually 4‒6 contractile vacuoles arranged in a single row extending along either right or left side of macronucleus, individual vacuoles very conspicuous because 7‒11 μm across during diastole; excretory pores observed neither in vivo nor after protargol impregnation (Figs. 7A, B, H–M, 8A‒C). Cortex flexible; not, or only slightly, furrowed by ciliary rows; no specific granules recognizable in vivo. Cytoplasm colorless, hyaline, contains some lipid droplets and innumerable granules (Fig. 8A‒F).

Somatic ciliature holotrichous and composed of monokinetids. Somatic cilia about 15 μm long in vivo, very narrowly spaced and arranged in about 20 meridional rows on ventral and 23 meridional rows on dorsal body side. Individual ciliary rows ordinarily spaced, i.e., interkinetal distance approximately 1.8 μm after protargol impregnation (Figs. 7B, E, 9B, C). Silverline system very loosely meshed, meshes relatively large and roughly rectangular; primary meridians connect kinetids within somatic kineties; short silverlines connect every fourth to fifteenth basal body of adjacent somatic kineties (Figs. 7F, 9A, D).

Family Maupasellidae Cépède, 1910

Genus *Buchneriella* Heidenreich, 1935 (type species: *Buchneriella criodrili* Heidenreich, 1935)

## *Buchneriella criodrili* Heidenreich, 1935 (Fig. 10)

**Fig. 10:**
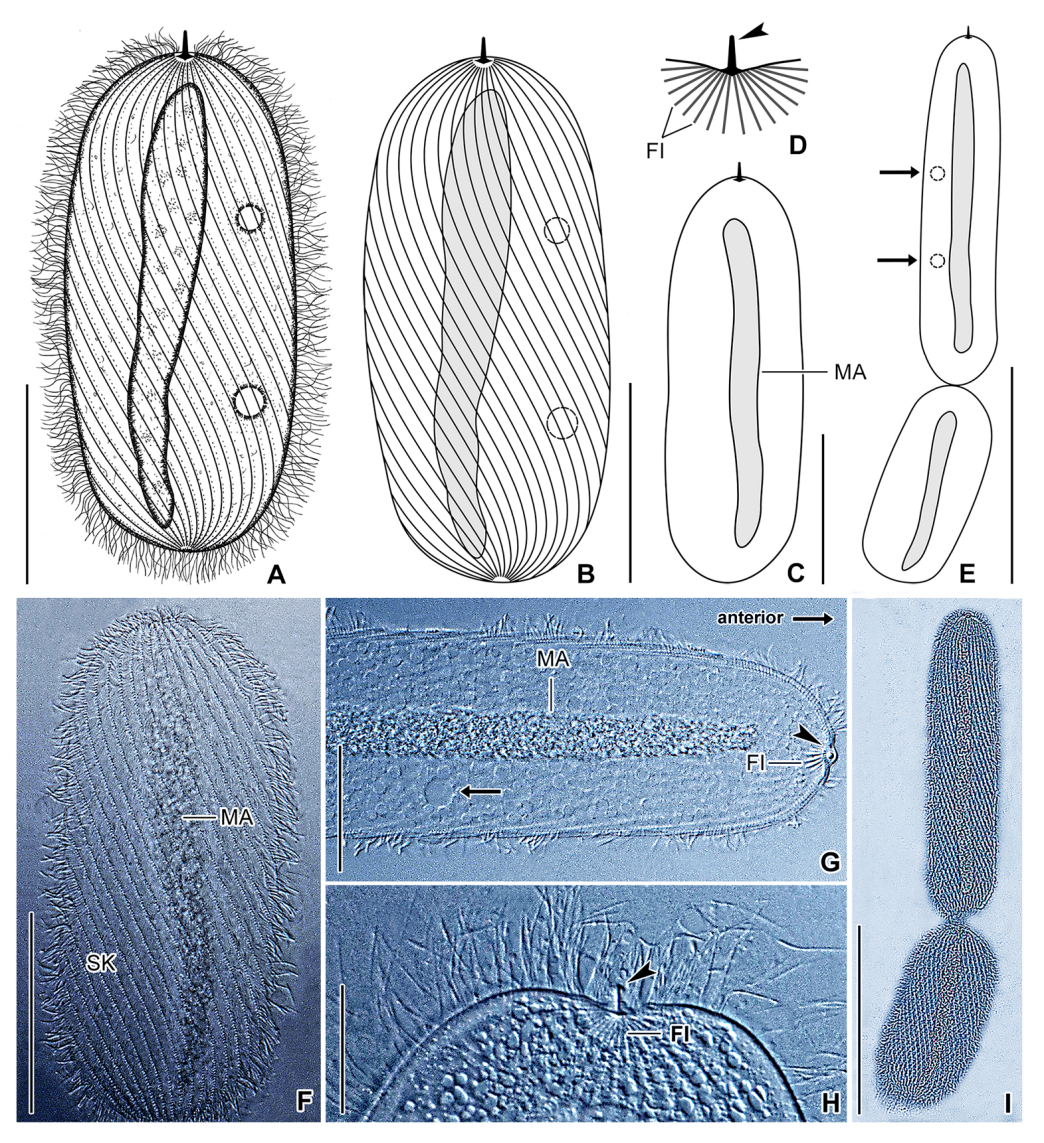
*Buchneriella criodrili* in vivo, neotype population (**A**‒**I**). **A**, **C**, **F** General body organization of representative specimens. **B** Semi-schematic diagram, showing the ciliary pattern, the nuclear and contractile vacuole apparatus, as well as the skeletal system. **D**, **H** Details of the skeletal apparatus. Arrowheads denote the thorn. **E**, **I** A very late divider. Arrows mark the contractile vacuoles. **G** Detail of the anterior body portion, showing the macronucleus, contractile vacuole (arrow), and fibers of the skeletal apparatus. The thorn (arrowhead) is curved backward forming a right angle with the ventral side.* FI* fibers, *MA* macronucleus, *SK* somatic kineties. Scale bars: 20 μm (**G**, **H**), 40 μm (**A**‒**C**, **F**), 100 μm (**E**, **I**)

1935 *Buchneriella criodrili* Heidenreich, 1935, Archiv für Protistenkunde 84: 349, Taf. 6 Fig. 11, Taf. 7 Figs. 13‒17 (detailed description with figures; type host: *Criodrilus lacuum*)
1952 *Buchneriella criodrili* Heidenreich ‒ de Puytorac, Bulletin de la Société zoologique de France 76: 332 (listed)
1957 *Buchneriella criodrili* Heidenreich 1935 ‒ de Puytorac, Archives de zoologie expérimentale et générale 94: 106 (listed)
1960 *Buchneriella criodrili* Heidenreich, 1935 ‒ de Puytorac, Journal of Protozoology 7: 279, Figs. 3, 5‒9, 11‒14 (description of a population from Lake Ohrid, with figures showing the detail of the skeletal apparatus and silverline system; hosts: *Criodrilus lacuum* and *C. ohridanum*)
1972 *Buchneriella criodrili* Heidenreich, 1935 ‒ de Puytorac, Protistologica 8: 32, Fig. 43 (taxonomic revision, microphotograph showing the silverline system, hosts: *Criodrilus lacuum* and *C. ohridanum*)

### Neotypification

We neotypify *B. criodrili* with a Slovak population from the Stupavský potok stream for the following objections: (1) the type locality is not mentioned in the original description (see Art. 75.3.1 of the ICZN 1999); (2) characters differentiating *B. criodrili* from related species are provided in ‘Improved diagnosis’ below (see Art. 75.3.2); (3) the neotype is properly labeled to ensure its recognition (see Art. 75.3.3); (4) it is generally known that no type material is available from the species described by Heidenreich (1935) (see Art. 75.3.4); (5) the neotype matches very well the original description; (6) the neotype is from the same biogeographic region (Palearctic) and type host (*C. lacuum*) (see Art. 75.3.6 and Foissner 2002); and (7) genomic DNA, as well as genetic data from the rDNA operon and two mitochondrial genes are available for the neotype and have been deposited in a recognized scientific institution (see Art. 75.3.7 and ‘Type material’ below) and in the GenBank database (https://www.ncbi.nlm.nih.gov/nucleotide/).

### Improved diagnosis (based on neotype population)

Body size 100‒200 × 30‒75 μm in vivo. Shape elliptical with both body ends rounded. Skeletal apparatus at cell apex and composed of a basal plate, a thorn, and 9‒16 supporting fibers. Macronucleus rod-like and ~ 85‒180 × 8‒12 μm in size. Two or three contractile vacuoles arranged in a single row. About 30‒40 bipolar and helically extending ciliary rows.

### Type locality

Not provided in the original description. The neotype is from the hyporeal of the Stupavský potok stream, Stupava, Slovakia, 48°16′38.2″N 17°02′25.0″E. According to Article 76.3 of the ICZN (1999), the place of origin of the neotype becomes the type locality.

### Type host

Heidenreich (1935) described *B*. *criodrili* from the intestinal tract of *Criodrilus lacuum*. Later, de Puytorac (1960) confirmed the type host *C. lacuum*. ND1 and COI sequences of the *C. lacuum* (ST 34) specimen carrying the neotype specimen of *B*. *criodrili* have been deposited in GenBank under the following accession numbers: PQ247166 and PQ243272, respectively.

### Type material

No material is available from the type population. A DNA sample of the neotype specimen (ST 77 CL) has been deposited in the Natural History Museum, Vajanského nábrežie 2, 810 06 Bratislava, Slovakia (ID Collection Code 01427563). The 18S rRNA gene, ITS1-5.8S-ITS2-28S rRNA gene, 16S rRNA gene, and cytochrome *c* oxidase subunit I sequences of the neotype specimen have been deposited in GenBank under the following accession nos. PQ238896, PQ240662, PQ249023, and PQ247159, respectively.

### Etymology

The species epithet is a singular genitive case of the Latin noun *Criodril*·*us*, -*i* [m], meaning a *Buchneriella* from *Criodrilus*. The species-group name is to be treated as an adjective used as a substantive in the genitive case, because of its derivation from the host’s generic name (Article 11.9.1.4. of the ICZN 1999).

### Description of neotype population

Body size about 100‒200 × 30‒75 μm in vivo, length:width ratio 2.6‒3.3:1; slightly dorso-ventrally flattened, not contractile. Body outline elliptical to narrowly elliptical, both body ends narrowly to broadly rounded (Fig. 10A‒C, F). Cell apex bears skeletal apparatus composed of a basal plate, a thorn, and 9‒16 supporting fibers. Basal plate about 3 μm wide in vivo, very broadly V-shaped, i.e., plate arms form an obtuse angle of 210°. Thorn inconspicuous because only ~ 4.5 μm long in vivo, fixed or mobile, directed forward or curved backward forming an acute or a right angle with ventral side. Supporting fibers radiate into cytoplasm from posterior side of basal plate in a fan-like pattern, about 4‒5 μm long in vivo (Fig. 10A‒D, G, H).

Macronucleus rod-like, about 85‒180 × 8‒12 μm in size in vivo, extends along main cell’s axis, begins about 11 μm apart from anterior body pole and terminates about 4‒12 μm ahead of posterior body pole, occupying ca. 90% of body length (Fig. 10A‒C, F, G). Micronucleus not observed. Two or three contractile vacuoles arranged in a single row: anterior vacuole at beginning of second body third, posterior vacuole at end of second body third, individual vacuoles about 5‒6 μm across during diastole; excretory pores not observed (Fig. 10A, B, G). Cortex flexible; not, or only slightly, furrowed by ciliary rows; no specific granules recognizable in vivo. Cytoplasm colorless, hyaline, contains innumerable granules; prokaryotic endosymbionts not observed (Fig. 10F‒H).

Somatic ciliature holotrichous and composed of monokinetids. Somatic cilia ~ 5‒7 μm long in vivo, very narrowly spaced and arranged in about 30‒40 rows. Ciliary rows helical and bipolar, i.e., commence at level of skeletal basal plate and terminate slightly ahead of posterior body pole, leaving a small blank field. Individual ciliary rows ordinarily spaced, i.e., interkinetal distance approximately 2 μm (Fig. 10A, B, F).

Only one very late divider was observed. The division occurred in freely motile (non-encysted) conditions. Binary fission was homothetogenic (i.e., the axes of the proter and the opisthe had the same orientation) and anisotomic without catenulation (i.e., asymmetric and without chain formation). Thus, the posterior daughter cell (opisthe) was distinctly smaller than the anterior daughter cell (proter) (94 × 45 μm vs. 165 × 36 μm) (Fig. 10E, I).

## Discussion

### Eco-evolutionary associations of astome ciliates with their annelid hosts

Members of the subclass Astomatia inhabit the digestive tract of planarians, mollusks, annelids (including polychaetes, oligochaetes, and leeches), and poikilotherm vertebrates (newts and frogs) (Lynn 2008). Molecular analyses have, however, revealed that mouthless ciliates classified in the Astomatia (Lynn 2008) do not form a monophyletic group, questioning the broad host spectrum. Mouthless ciliates inhabiting annelids form a monophylum that is separated from mouthless ciliates isolated from planarians or mollusks (Obert and Vďačný 2019, 2020b, 2021; Obert et al. 2021; Park and Leander 2024). The planarian-dwelling mouthless *Haptophrya* clusters within the subclass Scuticociliatia along with the free-living *Dexiotricha* and the symbiotic *Conchophthirus* (Antipa et al. 2020; Li et al. 2024; Rataj and Vďačný 2019; Rataj et al. 2022; Zhang and Vďačný 2024), while the mollusk-dwelling mouthless *Clausilocola* belongs to the subclass Hymenostomatia (Zhang and Vďačný 2022). The astome affinity of *Protoanoplophrya* and *Desmophrya*, which were discovered in gastropods and bivalves (Raabe 1932, 1933), awaits to be tested with molecular methods. Since there are no molecular data from mouthless ciliates infecting newts and frogs, their classification within the subclass Astomatia should be taken with caution (Rataj and Vďačný 2018; Rataj et al. 2022).

So far, only astomes associated with marine polychaetes and terrestrial earthworms have been studied with molecular tools (Fokam et al. 2011; Obert and Vďačný 2019, 2020b, 2021; Obert et al. 2021; Park and Leander 2024; Sauvadet et al. 2017). Polychaetes were suggested as the ancestral hosts of all astomes and endogeic earthworms as the ancestral hosts of astomes living in terrestrial habitats. To better understand the coevolution of astomes and annelids, ciliates from freshwater oligochaetes need to be added to phylogenetic analyses. We selected one representative from an old lumbriculid lineage belonging to the Lumbriculata clade and another representative from a more recent criodrilid lineage belonging to the Crassiclitellata clade (= true earthworms) (Erséus et al. 2020). By adding astomes from these two aquatic oligochaete lineages, we could test whether (1) astomes first colonized marine polychaetes, then freshwater lumbriculids, and finally aquatic and terrestrial crassiclitellates and (2) endogeic lumbricids were the first colonized terrestrial earthworms that spread astomes to epigeic and anecic earthworms.

Our SIMMAP reconstruction analyses revealed that the last common ancestor of astomes invaded the digestive tract of marine polychaetes ~ 428‒241 Ma, then freshwater lumbriculids were invaded ~ 345‒227 Ma, and finally terrestrial earthworms were colonized ~ 255‒200 Ma (Fig. 3A, B). These findings parallel those of annelid phylogeny. Polychaetes are a mostly marine paraphyletic assemblage that contains clitellates (i.e., oligochaetes and leeches), and the oldest stem-group polychaete fossils come from the Early Cambrian about 520 Ma (Conway Morris and Peel 2008). Astomes from marine polychaetes form at least two independent deep-branching lineages (represented here by the genera *Durchoniella* and *Pennarella*) that might have emerged ~ 100 Ma after the presence of polychaetes in the fossil record. As concerns phylogenetic relationships among clitellate oligochaetes, Lumbriculata (Lumbriculida + Hirudinea) are sister to the Crassiclitellata + Moniligastridae + Haplotaxidae clade and, according to phylogenomic analyses, these sister groups separated ~ 310 Ma (Erséus et al. 2020). Crassiclitellata comprise at least 21 families of true (mostly terrestrial) earthworms, including Lumbricidae, Criodrilidae, Almidae, Megascolecidae, and Glossoscolecidae (Anderson et al. 2017; Schmelz et al. 2021). Astomes isolated from lumbriculids branched off before the radiation of astomes inhabiting crassiclitellates. Lumbriculid- and crassiclitellate-dwelling astomes diverged ~ 300 Ma, matching the divergence of their host groups. Astomes associated with crassiclitellates form a monophylum whose radiation (255‒200 Ma) corresponds to the root age (261‒200 Ma) of the time-calibrated phylogeny of earthworms (Domínguez et al. 2015). Phylogenomic analyses revealed a deep split at the base of crassiclitellates into two major clades: one (comprising, for instance, Lumbricidae and Criodrilidae) with a largely Northern Hemisphere (Laurasian) distribution and the other one (comprising, e.g., Almidae, Glossoscolecidae, and Megascolecidae) with a primarily Southern Hemisphere (Gondwanan) distribution. The north–south split dates to ~ 161–185 Ma, supporting the role of the breakup of Pangaea during the Mesozoic in earthworm phylogeny and biogeography (Anderson et al. 2017). However, the north–south split of earthworms is not recognizable in the phylogeny of astomes, as almid- and megascolecid-dwelling astomes are not closely related and are nested within the paraphyletic group of lumbricid-dwelling astomes. This indicates that lumbricids served as a source of astomes for almid and megascolecid earthworms when paleocontinents made possible the occupation of South American, African, and Asian landmasses.

As concerns habitat evolution, annelids lived ancestrally in the sea where the majority of polychaetes still reside. On the other hand, the most recent common ancestor of clitellates colonized freshwater during the Devonian (419–359 Ma), and all major extant clitellate lineages arose over the next ~ 150 million years, with multiple lineages subsequently returning to marine habitats or invading land (Erséus et al. 2020). The endogeic lifestyle (subsoil-dwelling and geophagy) represents the ancestral life history of true earthworms, while epigeic (surface-dwelling and feeding on decomposing litter) and anecic (topsoil-dwelling and feeding on dead organic materials mixed with ingested soil) earthworms evolved several times convergently from endogeic ancestors (Domínguez et al. 2015). Phylogeny of astomes copies this habitat evolution of annelids. Deep-branching astomes were found in marine polychaetes, and our SIMMAP analyses also reconstructed the sea to be the ancestral habitat of astomes (Fig. 3B). Astomes colonized freshwater via lumbriculids in which they further radiated. Finally, astomes were brought to the land by crassiclitellates that ancestrally led an endogeic lifestyle (Domínguez et al. 2015; Erséus et al. 2020). Endogeic lumbricid earthworms served as a source of astomes for the aquatic criodrilids as well as for the endogeic almid and megascolecid earthworms (Fig. 3A, B). Mirroring the evolutionary history of life strategies of lumbricid earthworms, the transfer to epigeic and anecic earthworms happened multiple times independently (Fig. 3B). The host and the habitat evolution of astomes are correlated, indicating a tight bond of astomes with annelids both in space and time.

### Taxonomy and nomenclature of *Hoplitophrya secans*

Stein (1859) discovered *H. secans* in the digestive tract of *Saenuris variegata* and *Enchytraeus vermicularis*, and described it under the name *Opalina secans* without providing a figure and information about its type locality. Stein (1861) transferred *O*. *secans* to a newly established genus *Hoplitophrya*, corrected the name of its type host to *Lumbriculus variegatus*, and provided a brief description without a figure. Because the original description is incomplete, we based the identification of the neotype population on the authoritative re-description of de Puytorac (1972). Although most taxonomic features (body shape, skeletal and nuclear apparatus, contractile vacuole pattern, presence of cytoplasmic prokaryotic symbionts, and number of ciliary rows) match, the neotype population is more stumpy (200‒350 × 100‒130 μm) than the literature reports: 580 × 21 μm (Buchholz 1862), 225‒240 × 20‒30 μm (Kijenskij 1926), 285‒665 × 24‒32 μm (Rossolimo and Perzewa 1929), 280‒900 × 18‒45 μm (Meier 1954), and 240‒500 × 20‒60 μm (de Puytorac 1972). Molecular information from slender populations is needed to test whether *H. secans* has two forms, stumpy and slender. Figure 14 in Table 2 illustrated by de Puytorac (1972) suggests that the width differences might be attributed to the stage in the life cycle. Slender cells might be pre-dividers or derived from proters, while the stumpy cells might correspond to opisthe post-dividers. In this light, we consider all aforementioned populations to be conspecific.

Heidenreich (1935) and de Puytorac (1972) revised *H. secans* and proposed many synonyms. We support, however, only three of them: *Opalina lumbriculi* erected by Buchholz (1862), *Hoplitophrya lumbriculi* established by Kijenskij (1926), and *Protoradiophrya armata* discovered by Rossolimo and Perzewa (1929). On the other hand, we do not find *O. naidos* described by Lankester (1870), *O. spiculata* by Warpachowsky (1886), and *H. secans* sensu Bütschli (1889), Schewiakoff (1896), and Cépède (1910) to be conspecific with *H. secans*. *Opalina naidos*, which was discovered in *Nais serpentina* and *Lumbriculus* sp. from Hampstead, England by Lankester (1870), differs from *H. secans* by the lack (vs. presence) of a fibrillar spicule. Cépède (1910) suggested transferring *O. naidos* to *Anoplophrya*, which seems to be reasonable since this genus does not exhibit any fibrillar spicule. *Opalina spiculata*, described by Warpachowsky (1886), can be distinguished from *H. secans* by the longer fibrillar spicule (extending almost along the whole cell length vs. in the anterior body fifth). Bütschli (1889) reported *H. secans* from *Nais*. Its conspecificity with *H. secans* can be, however, excluded based on the anisotomic fission with catenulation, that is, chain formation, which has been never reported for the true *H. secans*. Schewiakoff (1896) found *H. secans* in *Lumbricus terrestris*, *Lumbriculus variegatus*, and *Enchytraeus vermicularis* and considered it to be conspecific with *O. spiculata* sensu Warpachowsky (1886). Such a broad host spectrum for one astome species is unlikely (Obert et al. 2021). Moreover, *Lumbricus terrestris* is phylogenetically distant from and has very different ecological demands than *Lumbriculus variegatus*. Cépède’s (1910) species is likely also a misidentified *H. secans* because its fibrillar spicule extends behind the mid-body (vs. terminates in the anterior body fifth). The heterogeneous host spectrum (*Lumbricus terrestris* and *Lumbriculus variegatus*) reported for *H. secans* by Cépède (1910) is doubtful as well. Cépède (1910) transferred *H*. *secans* to the genus “*Mesnillella*,” which is an incorrect subsequent spelling of *Mesnilella* and therefore unavailable according to Articles 33.3 and 33.5 of the ICZN (1999). He also assigned *O. spiculata* to the genus *Mesnilella* and synonymized it with *H*. *secans*. The combination of *H. secans* with *Mesnilella* was, however, not accepted by subsequent revising authors (de Puytorac 1972; Heidenreich 1935), as both genera conspicuously differ in the architecture of their skeletal apparatuses.

### Taxonomy and nomenclature of *Mesnilella clavata*

*Mesnilella clavata* was discovered by Leidy (1855) in *Lumbriculus tenuis* (species inquirenda) and established under the name *Leucophrys clavata* without any description or figure. Stein (1861) stated that *L. tenuis* sensu Leidy (1855) is *L. variegatus*, and erected *Hoplitophrya securiformis* for a ciliate isolated from *L. variegatus*, providing a brief description but no figure. Kent (1881) also mentioned *H*. *securiformis* from *L. variegatus*, again with a brief description and without a figure. Bütschli (1889) gave the first illustration of *H. clavata* and proposed *H*. *securiformis* to be its junior synonym. Cépède (1910) accepted this synonymization and established a new genus, *Mesnilella*, for *H. clavata*. He also proposed that *Anoplophrya* (?) *clavata* isolated from *Lumbriculus limosus* might be conspecific with *M*. *clavata*, which was repeated by Cheissin (1930). Schuster (1912), however, overlooked Cépède’s (1910) work and transferred *H. clavata* to a new genus, *Plagiophrya* Schuster, 1912, which was not recognized by subsequent revisers (Cheissin 1930; de Puytorac 1972; Heidenreich 1935; Kijenskij 1926; Penard 1922).

All reliable reports of *M. clavata* come exclusively from *L. variegatus* (Cépède 1910; de Puytorac 1972; Heidenreich 1935; Kijenskij 1926; Mackinnon and Adam 1924; Meier 1954; Penard 1922; Schewiakoff 1896; Schuster 1912). The available descriptions are consistent, matching our observations very well (Bütschli 1889; Cépède 1910; de Puytorac 1972; Heidenreich 1935; Kaczanowski 1965; Kent 1881; Kijenskij 1926; Mackinnon and Adam 1924; Penard 1922; Schewiakoff 1896). Besides the typical stumpy form, Heidenreich (1935) mentioned huge variability in body size. Some specimens were not longer than 90 μm (very likely post-dividers), while other individuals were 150‒240 μm long. De Puytorac (1972) also reported a much longer and slender variety (225 × 15 μm). Whether these size differences are population-specific, depend on life stage, and/or just reflect phenotypic plasticity, needs to be addressed using detailed morphological observations and multigene data from the larger and slender forms. We did not observe the longer and slender variety either in the neotype population from the Jurské jazierko pond or from Kráľovská lúka in the riparian zone of the River Danube.

### Taxonomy and nomenclature of *Buchneriella criodrili*

Besides the original description by Heidenreich (1935), *B. criodrili* was mentioned only a few times in the literature (de Puytorac 1952, 1957, 1960, 1972). We have rediscovered *B. criodrili* after more than a half of century, indicating that it is a rare species that may sometimes co-occur with *Maupasella criodrili* (de Puytorac 1960). All reports including the present one come consistently from *C. lacuum*. In addition, de Puytorac (1960) isolated *B. criodrili* also from *C. ohridanum*, which is suggestive of high structural and phylogenetic host specificity. Nevertheless, the conspecificity of *Buchneriella* populations isolated from different *Criodrilus* species needs to be confirmed by multi-gene data.

The neotype population matches those studied by Heidenreich (1935) and de Puytorac (1960, 1972) in body shape and size (100‒200 × 30‒75 μm in neotype specimens, 110‒220 × 22‒28 μm according to Heidenreich 1935, 140‒160 × 40‒50 μm according to de Puytorac 1972), the skeletal and nuclear apparatus as well as in the contractile vacuole and somatic ciliary pattern. The variability in body width most likely reflects the stage in the life cycle. The much more slender cells are derived from proters, while the stumpy cells from opisthes (Fig. 10E, I).

Our neotypification will promote stability in the use of the name *B. criodrili* especially when further congeners are discovered in various criodrilids in the Oriental biogeographic region. Hitherto, only one other congener, *B. pheretimae* de Puytorac and Rakotoarivelo, 1965, was described from the megascolecid earthworm *Pheretima* sp. by de Puytorac and Rakotoarivelo (1965). Besides the host earthworm, *B. pheretimae* differs from *B. criodrili* by a much larger body (270‒500 × 45‒50 μm vs. 100‒220 × 22‒75 μm) and a higher number of ciliary rows (59‒60 vs. 36‒42).

## Conclusions

Our comparative phylogenetic network analyses suggested that: (1) astomes evolved in the sea during the Paleozoic and their ancestral hosts were polychaetes, then they were brought to freshwater where they colonized lumbriculids, and finally reached terrestrial habitats in tandem with their true earthworm hosts; (2) lumbricid earthworms likely spread astomes to criodrilid, almid, and megascolecid earthworms before the breakup of Pangaea in the Mesozoic; (3) the ancestral host of the earthworm-dwelling astome clade led an endogeic lifestyle; (4) there were multiple independent transfers of astomes from endogeic to epigeic and anecic as well as back to (semi)aquatic earthworms such as criodrilids; and (5) host and the habitat evolution of astomes are correlated, indicating a tight bond of astomes with annelids both in space and time.

## Supplementary information

Below is the link to the electronic supplementary material.

Supplementary file1 (PDF 614 KB)

## Data Availability

All data generated or analyzed during this study are included in this published article and supplementary material and can be found in online repositories. The names of the repositories and accession numbers can be found at: https://www.ncbi.nlm.nih.gov/genbank/.
