# Supplementary material for: Rediscovery and morpho-molecular characterization of three astome ciliates, with new insights into eco-evolutionary associations of astomes with their annelid hosts
Source: Mar Life Sci Technol. 2025 Mar 17;7(2):231–55. doi: 10.1007/s42995-024-00275-5 (PMC12102460; doi:10.1007/s42995-024-00275-5)
Supplement: Supplementary file 1 — Supplementary file1 (PDF 614 KB) [file 42995_2024_275_MOESM1_ESM.pdf]

SUPPLEMENTARY MATERIAL

Rediscovery and morpho-molecular characterization of three astome ciliates, with new insights into eco-evolutionary associations of astomes with their annelid hosts

Tomáš Obert[1] · Tengyue Zhang[2] · Ivan Rurik[1] · Peter Vďačný[1]

[1] Department of Zoology, Faculty of Natural Sciences, Comenius University in Bratislava, 842 15 Bratislava, Slovak Republic

[2] The Key Laboratory of Zoological Systematics and Application, College of Life Sciences, Hebei University, Baoding 071002, China

✉ Peter Vďačný
peter.vdacny@uniba.sk

Pages: **9**

Figures: **2**

Tables: **3**

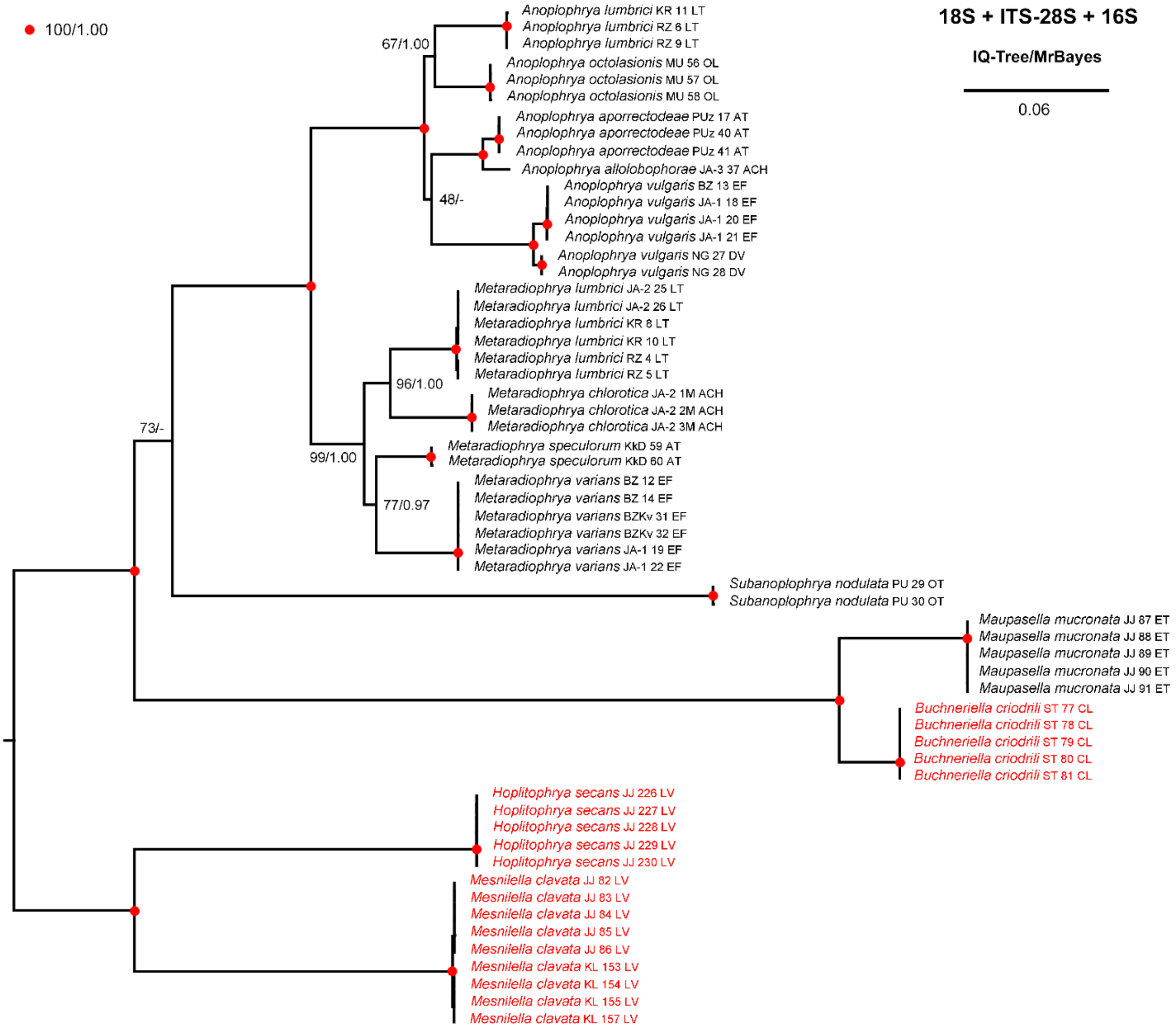

Supplementary Fig. S1. Phylogenetic tree of the subclass Astomatia based on the nuclear 18S rRNA and ITS-28S rRNA gene sequences as well as the mitochondrial 16S rRNA gene sequences, showing the systematic positions of astome ciliates isolated from *Lumbriculus variegatus* and *Criodrilus lacuum*. Bootstrap values for maximum likelihood conducted in IQ-TREE and posterior probabilities for Bayesian inferences conducted in MrBayes were mapped onto the best scoring IQ-Tree. Sequences marked in red were obtained during this study. Fully statistically supported nodes are marked with red solid circles. Dash (–) indicates a mismatch in tree topologies. The scale bar denotes six substitutions per one hundred nucleotide positions

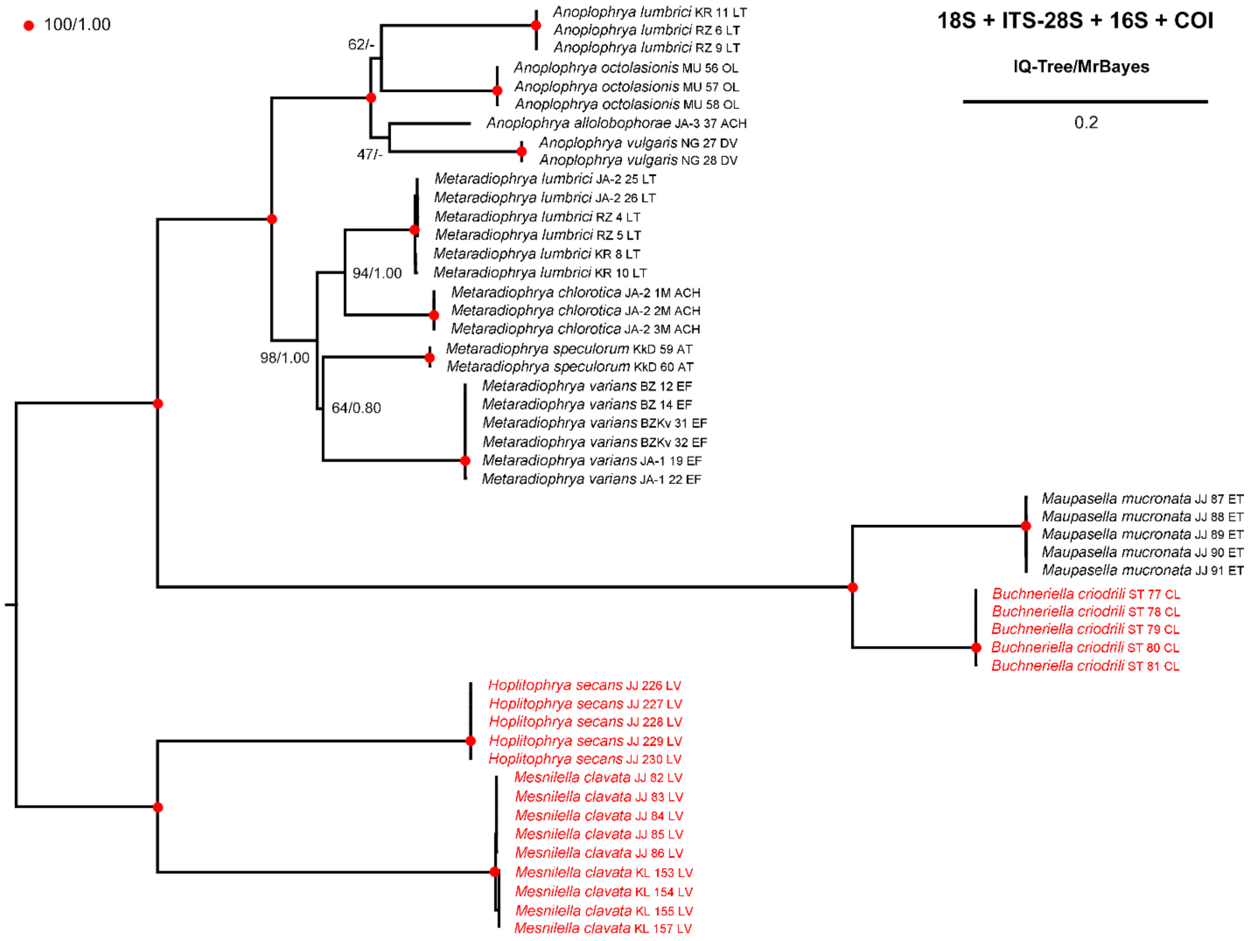

Supplementary Fig. S2. Phylogenetic tree of the subclass Astomatia based on the nuclear 18S rRNA and ITS-28S rRNA gene sequences as well as the mitochondrial 16S rRNA gene and COI sequences, showing the systematic positions of astome ciliates isolated from *Lumbriculus variegatus* and *Criodrilus lacuum*. Bootstrap values for maximum likelihood conducted in IQ-TREE and posterior probabilities for Bayesian inferences conducted in MrBayes were mapped onto the best scoring IQ-Tree. Sequences marked in red were obtained during this study. Fully statistically supported nodes are marked with red solid circles. Dash (–) indicates a mismatch in tree topologies. The scale bar denotes two substitutions per ten nucleotide positions

Supplementary Table S1. List of taxa with GenBank accession numbers of corresponding mitochondrial 18S rRNA gene sequences included in dataset 1.

Taxon	GeBank	Taxon	GeBank	Taxon	GeBank
**Astomatia**		**Astomatia**		**Hymenostomatia**	
Almophrya bivacuolata	HQ446281	*Mesnilella clavata* KL 154 LV	PQ238893	*Tetrahymena australis*	X56167
Anoplophrya allolobophorae JA-3 37 ACH	MZ048824	*Mesnilella clavata* KL 155 LV	PQ238894	*Tetrahymena borealis*	M98020
Anoplophrya aporrectodeae PUz 17 AT	MZ048825	*Mesnilella clavata* KL 157 LV	PQ238895	*Tetrahymena hegewischi*	X56166
Anoplophrya aporrectodeae PUz 40 AT	MZ048826	*Metaracoelophrya intermedia*	HQ446278	*Tetrahymena rostrata*	AF364042
Anoplophrya aporrectodeae PUz 41 AT	MZ048827	*Metaracoelophrya* sp. 1	HQ446277	**Peniculia**	
Anoplophrya lumbrici KR 11 LT	MN121061	*Metaracoelophrya* sp. 2	HQ446279	*Paramecium tetraurelia*	X03772
Anoplophrya lumbrici KR 9 LT	MZ048831	*Metaradiophrya chlorotica* JA-2 1M ACH	MZ048835	*Frontonia vernalis*	U97110
Anoplophrya lumbrici RZ 6 LT	MN121062	*Metaradiophrya chlorotica* JA-2 2M ACH	MZ048836	*Lembadion bullinum*	AF255358
Anoplophrya octolasionis MU 56 OL	MZ048828	*Metaradiophrya chlorotica* JA-2 3M ACH	MZ048837	**Peritrichia**	
Anoplophrya octolasionis MU 57 OL	MZ048829	*Metaradiophrya lumbrici* JA-2 25 LT	MN121068	*Campanella umbellaria*	AF401524
Anoplophrya octolasionis MU 58 OL	MZ048830	*Metaradiophrya lumbrici* JA-2 26 LT	MN121069	*Carchesium polypinum*	AF401522
Anoplophrya vulgaris BZ 13 EF	MZ048834	*Metaradiophrya lumbrici* KR 8 LT	MN121070	*Epicarchesium abrae*	DQ190462
Anoplophrya vulgaris JA-1 18 EF	MZ048832	*Metaradiophrya lumbrici* KR 10/1 LT	MN121071	*Epistylis galea*	AF401527
Anoplophrya vulgaris JA-1 20 EF	MN121065	*Metaradiophrya lumbrici* RZ 4 LT	MN121074	*Epistylis plicatilis*	AF335517
Anoplophrya vulgaris JA-1 21 EF	MN121066	*Metaradiophrya lumbrici* RZ 5 LT	MN121075	*Pseudovorticella punctata*	DQ190466
Anoplophrya vulgaris NG 27 DV	MZ048833	*Metaradiophrya* sp.	HQ446279	*Vorticella campanula*	AF335518
Anoplophrya vulgaris NG 28 DV	MN121067	*Metaradiophrya speculorum* HkD 59 AT	MW182012	*Vorticella microstoma*	DQ868347
Buchneriella criodrili ST 77 CL	PQ238896	*Metaradiophrya speculorum* HkD 60 AT	MW182013	*Zoothamnium arbuscula*	AF401523
Buchneriella criodrili ST 78 CL	PQ238897	*Metaradiophrya varians* BZ 12 EF	MN121076	*Zoothamnium pluma*	DQ662854
Buchneriella criodrili ST 79 CL	PQ238898	*Metaradiophrya varians* BZ 14 EF	MN121077	**Scuticociliatia**	
Buchneriella criodrili ST 80 CL	PQ238899	*Metaradiophrya varians* BZkv 31 EF	MZ048838	*Ancistrum crassum*	HM236340
Buchneriella criodrili ST 81 CL	PQ238900	*Metaradiophrya varians* BZkv 32 EF	MZ048839	*Boveria subcylindrica*	FJ848878
Durchoniella brasili	FN998990	*Metaradiophrya varians* JA-1 19 EF	MN121078	*Cinetochilum ovale*	FJ870103
Durchoniella legeriduboscqui	FN998995	*Metaradiophrya varians* JA-1 22 EF	MN121079	*Conchophthirus acuminatus*	OR127221
Durchoniella sp.	FN998993	*Njinella prolifera*	HQ446276	*Conchophthirus curtus*	OR127229
Eudrilophrya complanata	HQ446280	*Paraclausilocola constricta*	HQ446275	*Conchophthirus unionis*	OR127243

Taxon	GeBank	Taxon	GeBank	Taxon	GeBank
Hoplitophrya secans JJ 226 LV	PQ238882	*Paraclausilocola elongata*	HQ446274	*Cyclidium glaucoma*	Z22879
Hoplitophrya secans JJ 227 LV	PQ238883	*Pennarella elegantia*	PP109353	*Dexiotricha granulosa*	KF878931
Hoplitophrya secans JJ 228 LV	PQ238884	*Subanoplophrya nodulata* PU 29 OT	MN121063	*Dexiotricha* sp. 1	KF878932
Hoplitophrya secans JJ 229 LV	PQ238885	*Subanoplophrya nodulata* PU 30 OT	MN121064	*Haptophrya dugesiarum*	OL752521
Hoplitophrya secans JJ 230 LV	PQ238886	**Apostomatia**		*Haptophrya planariarum*	OL752480
Maupasella mucronata KDo 33 ET	MW182008	*Gymnodinioides pitelkae*	EU503534	*Haptophrya schmidtearum*	OL752526
Maupasella mucronata KDo 34 ET	MW182009	*Hyalophysa lwoffi*	EU503538	*Philaster apodigitiformis*	FJ648350
Maupasella mucronata KDo 35 ET	MW182010	*Vampyrophrya pelagica*	EU503539	*Philaster sinensis*	KJ815049
Maupasella mucronata KDo 36 ET	MW182011	**Hymenostomatia**		*Philasterides armatalis*	FJ848877
Mesnilella clavata JJ 82 LV	PQ238887	*Bromeliophrya brasiliensis*	AJ810075	*Pleuronema coronatum*	AY103188
Mesnilella clavata JJ 83 LV	PQ238888	*Colpidium campylum*	X56532	*Pleuronema setigerum*	FJ848874
Mesnilella clavata JJ 84 LV	PQ238889	*Glaucoma chattoni*	X56533	*Uronema marinum*	GQ465466
Mesnilella clavata JJ 85 LV	PQ238890	*Glaucomides bromelicola*	AJ810077	*Uronemella filificum*	EF486866
Mesnilella clavata JJ 86 LV	PQ238891	*Ichthyophthirius multifiliis*	U17354		
Mesnilella clavata KL 153 LV	PQ238892	*Ophryoglena catenula*	U17355		

Supplementary Table S2. Characterization and origin of nuclear and mitochondrial sequences of astome ciliates included in datasets 2 and 3.

Species	Specimen	Host species	Locality[a]	18S rRNA gene	ITS region and 28S rRNA gene	16S rRNA gene	COI gene
Anoplophrya allolobophorae	JA-3 37 ACH	*Allolobophora chlorotica*	JA-3	MZ048824	MZ048775	MZ048789	MZ044303
Anoplophrya aporrectodeae	PUz 17 AT	*Aporrectodea tuberculata*	PUz	MZ048825	MZ048776	MZ048790	–
	PUz 40 AT	*Aporrectodea tuberculata*	PUz	MZ048826	MZ048777	MZ048791	–
	PUz 41 AT	*Aporrectodea tuberculata*	PUz	MZ048827	MZ048778	MZ048792	–
Anoplophrya octolasionis	MU 56 OL	*Octolasion lacteovicinum*	MU	MZ048828	MZ048779	MZ048793	MZ044304
	MU 57 OL	*Octolasion lacteovicinum*	MU	MZ048829	MZ048780	MZ048794	MZ044305
	MU 58 OL	*Octolasion lacteovicinum*	MU	MZ048830	MZ048781	MZ048795	MZ044306
Anoplophrya lumbrici	KR 9 LT	*Lumricus terrestris*	KR	MZ048831	MZ048782	MZ048796	MZ044307
	KR 11 LT	*Lumricus terrestris*	KR	MN121061	MN897871	MZ048797	MZ044308
	RZ 6 LT	*Lumricus terrestris*	RZ	MN121062	MN897872	MZ048798	MZ044309
Anoplophrya vulgaris	JA-1 18 EF	*Eisenia andrei*	JA-1	MZ048832	MZ048783	MZ048799	–
	JA-1 20 EF	*Eisenia andrei*	JA-1	MN121065	MN897875	MZ048800	–
	JA-1 21 EF	*Eisenia andrei*	JA-1	MN121066	MN897876	MZ048801	–
	NG 27 DV	*Dendrobaena veneta*	NG	MZ048833	MN897877	MZ048802	MZ044310
	NG 28 DV	*Dendrobaena veneta*	NG	MN121067	MN897878	MZ048803	MZ044311
	BZ 13 EF	*Eisenia andrei*	BZ	MZ048834	MN897879	MZ048804	–
Buchneriella criodrili	ST 77 CL	*Criodrilus lacuum*	ST	PQ238896	PQ240662	PQ249023	PQ247159
Buchneriella criodrili	ST 78 CL	*Criodrilus lacuum*	ST	PQ238897	PQ240663	PQ249024	PQ247160
Buchneriella criodrili	ST 79 CL	*Criodrilus lacuum*	ST	PQ238898	PQ240664	PQ249025	PQ247161
Buchneriella criodrili	ST 80 CL	*Criodrilus lacuum*	ST	PQ238899	PQ240665	PQ249026	PQ247162
Buchneriella criodrili	ST 81 CL	*Criodrilus lacuum*	ST	PQ238900	PQ240666	PQ249027	PQ247163
Hoplitophrya secans	JJ 226 LV	*Lumbriculus variegatus*	JJ	PQ238882	PQ240648	PQ249009	PQ247145
Hoplitophrya secans	JJ 227 LV	*Lumbriculus variegatus*	JJ	PQ238883	PQ240649	PQ249010	PQ247146

Species	Specimen	Host species	Locality[a]	18S rRNA gene	ITS region and 28S rRNA gene	16S rRNA gene	COI gene
Hoplitophrya secans	JJ 228 LV	*Lumbriculus variegatus*	JJ	PQ238884	PQ240650	PQ249011	PQ247147
Hoplitophrya secans	JJ 229 LV	*Lumbriculus variegatus*	JJ	PQ238885	PQ240651	PQ249012	PQ247148
Hoplitophrya secans	JJ 230 LV	*Lumbriculus variegatus*	JJ	PQ238886	PQ240652	PQ249013	PQ247149
Mesnilella clavata	JJ 82 LV	*Lumbriculus variegatus*	JJ	PQ238887	PQ240653	PQ249014	PQ247150
Mesnilella clavata	JJ 83 LV	*Lumbriculus variegatus*	JJ	PQ238888	PQ240654	PQ249015	PQ247151
Mesnilella clavata	JJ 84 LV	*Lumbriculus variegatus*	JJ	PQ238889	PQ240655	PQ249016	PQ247152
Mesnilella clavata	JJ 85 LV	*Lumbriculus variegatus*	JJ	PQ238890	PQ240656	PQ249017	PQ247153
Mesnilella clavata	JJ 86 LV	*Lumbriculus variegatus*	JJ	PQ238891	PQ240657	PQ249018	PQ247154
Mesnilella clavata	KL 153 LV	*Lumbriculus variegatus*	KL	PQ238892	PQ240658	PQ249019	PQ247155
Mesnilella clavata	KL 154 LV	*Lumbriculus variegatus*	KL	PQ238893	PQ240659	PQ249020	PQ247156
Mesnilella clavata	KL 155 LV	*Lumbriculus variegatus*	KL	PQ238894	PQ240660	PQ249021	PQ247157
Mesnilella clavata	KL 157 LV	*Lumbriculus variegatus*	KL	PQ238895	PQ240661	PQ249022	PQ247158
Maupasella mucronata	KDo 33 ET	*Eiseniella tetraedra*	KDo	MW182008	MW181992	–	MZ044312
	KDo 34 ET	*Eiseniella tetraedra*	KDo	MW182009	MW181993	–	MZ044313
	KDo 35 ET	*Eiseniella tetraedra*	KDo	MW182010	MW181994	–	MZ044314
	KDo 36 ET	*Eiseniella tetraedra*	KDo	MW182011	MW181995	–	MZ044315
Meteradiophrya chlorotica	JA-2 1M ACH	*Allolobophora chlorotica*	JA-2	MZ048835	MZ048784	MZ048805	MZ044316
	JA-2 2M ACH	*Allolobophora chlorotica*	JA-2	MZ048836	MZ048785	MZ048806	MZ044317
	JA-2 3M ACH	*Allolobophora chlorotica*	JA-2	MZ048837	MZ048786	MZ048807	MZ044318
Metaradiophrya lumbrici	JA-2 25 LT	*Lumricus terrestris*	JA-2	MN121068	MN897880	MZ048808	MZ044319
	JA-2 26 LT	*Lumricus terrestris*	JA-2	MN121069	MN897881	MZ048809	MZ044320
	KR 8 LT	*Lumricus terrestris*	KR	MN121070	MN897882	MZ048810	MZ044321
	KR 10/1 LT	*Lumricus terrestris*	KR	MN121071	MN897883	MZ048811	MZ044322
	RZ 4 LT	*Lumricus terrestris*	RZ	MN121074	MN897884	MZ048812	MZ044323

Species	Specimen	Host species	Locality[a]	18S rRNA gene	ITS region and 28S rRNA gene	16S rRNA gene	COI gene
	RZ 5 LT	*Lumricus terrestris*	RZ	MN121075	MN897885	MZ048813	MZ044324
Metaradiophrya varians	BZ 12 EF	*Eisenia andrei*	BZ	MN121076	MN897886	MZ048814	MZ044325
	BZ 14 EF	*Eisenia andrei*	BZ	MN121077	MN897887	MZ048815	MZ044326
	JA-1 19 EF	*Eisenia andrei*	JA-1	MN121078	MN897888	MZ048816	MZ044327
	JA-1 22 EF	*Eisenia andrei*	JA-1	MN121079	MN897889	MZ048817	MZ044328
	BZkv 31 EF	*Eisenia andrei*	BZkv	MZ048838	MZ048787	MZ048818	MZ044329
	BZkv 32 EF	*Eisenia andrei*	BZkv	MZ048839	MZ048788	MZ048819	MZ044330
Metaradiophrya speculorum	HkD 59 AT	*Aporrectodea tuberculata*	HkD	MW182012	MW181996	MZ048820	MZ044331
	HkD 60 AT	*Aporrectodea tuberculata*	HkD	MW182013	MW181997	MZ048821	MZ044332
Subanoplophrya nodulata	PU 29 OT	*Octolasion tyrtaeum*	PU	MN121063	MN897873	MZ048822	–
	PU 30 OT	*Octolasion tyrtaeum*	PU	MN121064	MN897874	MZ048823	–

[a] For locality codes and further details, see Obert et al. (2021, 2022).

Supplementary Table S3. Characterization of newly obtained nuclear and mitochondrial sequences of astome ciliates.

Species	Specimen	Host species	Locality	18S rRNA gene	ITS region and 28S rRNA gene	16S rRNA gene	COI gene
Hoplitophrya secans	JJ 226 LV	*Lumbriculus variegatus*	Jurské jazierko	PQ238882	PQ240648	PQ249009	PQ247145
Hoplitophrya secans	JJ 227 LV	*Lumbriculus variegatus*	Jurské jazierko	PQ238883	PQ240649	PQ249010	PQ247146
Hoplitophrya secans	JJ 228 LV	*Lumbriculus variegatus*	Jurské jazierko	PQ238884	PQ240650	PQ249011	PQ247147
Hoplitophrya secans	JJ 229 LV	*Lumbriculus variegatus*	Jurské jazierko	PQ238885	PQ240651	PQ249012	PQ247148
Hoplitophrya secans	JJ 230 LV	*Lumbriculus variegatus*	Jurské jazierko	PQ238886	PQ240652	PQ249013	PQ247149
Mesnilella clavata	JJ 82 LV	*Lumbriculus variegatus*	Jurské jazierko	PQ238887	PQ240653	PQ249014	PQ247150
Mesnilella clavata	JJ 83 LV	*Lumbriculus variegatus*	Jurské jazierko	PQ238888	PQ240654	PQ249015	PQ247151
Mesnilella clavata	JJ 84 LV	*Lumbriculus variegatus*	Jurské jazierko	PQ238889	PQ240655	PQ249016	PQ247152
Mesnilella clavata	JJ 85 LV	*Lumbriculus variegatus*	Jurské jazierko	PQ238890	PQ240656	PQ249017	PQ247153
Mesnilella clavata	JJ 86 LV	*Lumbriculus variegatus*	Jurské jazierko	PQ238891	PQ240657	PQ249018	PQ247154
Mesnilella clavata	KL 153 LV	*Lumbriculus variegatus*	Kráľovská lúka	PQ238892	PQ240658	PQ249019	PQ247155
Mesnilella clavata	KL 154 LV	*Lumbriculus variegatus*	Kráľovská lúka	PQ238893	PQ240659	PQ249020	PQ247156
Mesnilella clavata	KL 155 LV	*Lumbriculus variegatus*	Kráľovská lúka	PQ238894	PQ240660	PQ249021	PQ247157
Mesnilella clavata	KL 157 LV	*Lumbriculus variegatus*	Kráľovská lúka	PQ238895	PQ240661	PQ249022	PQ247158
Buchneriella criodrili	ST 77 CL	*Criodrilus lacuum*	Stupavský potok	PQ238896	PQ240662	PQ249023	PQ247159
Buchneriella criodrili	ST 78 CL	*Criodrilus lacuum*	Stupavský potok	PQ238897	PQ240663	PQ249024	PQ247160
Buchneriella criodrili	ST 79 CL	*Criodrilus lacuum*	Stupavský potok	PQ238898	PQ240664	PQ249025	PQ247161
Buchneriella criodrili	ST 80 CL	*Criodrilus lacuum*	Stupavský potok	PQ238899	PQ240665	PQ249026	PQ247162
Buchneriella criodrili	ST 81 CL	*Criodrilus lacuum*	Stupavský potok	PQ238900	PQ240666	PQ249027	PQ247163
